# The gut microbiome in sickle cell disease: Characterization and potential implications

**DOI:** 10.1371/journal.pone.0255956

**Published:** 2021-08-25

**Authors:** Hassan Brim, James Taylor, Muneer Abbas, Kimberly Vilmenay, Mohammad Daremipouran, Sudhir Varma, Edward Lee, Betty Pace, Waogwende L. Song-Naba, Kalpna Gupta, Sergei Nekhai, Patricia O’Neil, Hassan Ashktorab

**Affiliations:** 1 Department of Pathology, Department of Medicine, Cancer Center, Microbiology and Center for Sickle Cell Disease, Howard University College of Medicine, Washington, DC, United States of America; 2 Hithru Analytics, Laurel, MD, United States of America; 3 University of Augusta, Augusta, GA, United States of America; 4 Division of Hematology, Oncology and Transplantation, Department of Medicine, University of Minnesota, Minneapolis, MN, United States of America; 5 Hematology/Oncology, Department of Medicine, University of California Irvine, Irvine, CA, United States of America; 6 Southern California Institute for Research and Education, Long Beach VA Healthcare System, Long Beach, CA, United States of America; 7 Food and Drug Administration, Silver Spring, MD, United States of America; University of Maine, UNITED STATES

## Abstract

**Background:**

Sickle Cell Disease (SCD) is an inherited blood disorder that leads to hemolytic anemia, pain, organ damage and early mortality. It is characterized by polymerized deoxygenated hemoglobin, rigid sickle red blood cells and vaso-occlusive crises (VOC). Recurrent hypoxia-reperfusion injury in the gut of SCD patients could increase tissue injury, permeability, and bacterial translocation. In this context, the gut microbiome, a major player in health and disease, might have significant impact. This study sought to characterize the gut microbiome in SCD.

**Methods:**

Stool and saliva samples were collected from healthy controls (n = 14) and SCD subjects (n = 14). Stool samples were also collected from humanized SCD murine models including Berk, Townes and corresponding control mice. Amplified 16S rDNA was used for bacterial composition analysis using Next Generation Sequencing (NGS). Pairwise group analyses established differential bacterial groups at many taxonomy levels. Bacterial group abundance and differentials were established using DeSeq software.

**Results:**

A major dysbiosis was observed in SCD patients. The Firmicutes/Bacteroidetes ratio was lower in these patients. The following bacterial families were more abundant in SCD patients: Acetobacteraceae, Acidaminococcaceae, Candidatus Saccharibacteria, Peptostreptococcaceae, Bifidobacteriaceae, Veillonellaceae, Actinomycetaceae, Clostridiales, Bacteroidacbactereae and Fusobacteriaceae. This dysbiosis translated into 420 different operational taxonomic units (OTUs). Townes SCD mice also displayed gut microbiome dysbiosis as seen in human SCD.

**Conclusion:**

A major dysbiosis was observed in SCD patients for bacteria that are known strong pro-inflammatory triggers. The Townes mouse showed dysbiosis as well and might serve as a good model to study gut microbiome modulation and its impact on SCD pathophysiology.

## Introduction

Sickle cell disease (SCD) is an inherited blood disorder due to mutations that arose in Africa in the *HBB* gene encoding the β-globin subunit of hemoglobin. SCD affects approximately 100,000 Americans and occurs in 1 out of every 365 births among African Americans in the US, while there are more than 300,000 annual SCD births worldwide [1]. Sickle hemoglobin mutations affect red blood cell function and shape which leads to a wide spectrum of manifestations that can vary in severity. Disease manifestations can be acute or chronic, and for a subset of patients they are associated with frequent hospitalizations due to repetitive and unpredictable painful episodes of vaso-occlusive crises (VOC). Indeed, sickled red blood cells (RBCs) have altered shape and rigidity due to the polymerization of deoxygenated mutant hemoglobins, making them susceptible to hemolysis. Rigid sickle RBCs occlude capillaries and venules leading to VOC, impaired blood and oxygen supply to organs and end organ damage. Hydroxyurea, L-glutamine, Crizanlizumab, Voxelotor and chronic transfusion therapy are available treatments to mitigate hemolysis or vaso-occlusive complications, while stem cell transplantation remains experimental, but potentially curative [2]. While the mitigation of VOC pain remains a major cause of hospitalizations, antibiotic prophylaxis prevents infections due to functional asplenia in SCD, leading to a significant reduction of early childhood mortality [2]. However, antibiotic administration for the first 5 years of life could alter gut microbiome diversity and composition due to both antibiotic effects and subclinical bowel ischemia from SCD [3].

The gut microbiome is the most consequential microbiome in our body [4]. Multiple studies highlighted its central role in many systemic processes that can affect some of the pathophysiological features of SCD. Indeed, the gut microbiome is a major player in metabolism, in gut-brain axis and systemic and local immunity [5–8]. It also plays an important role in the maintenance of the gut epithelial integrity. When such integrity is disrupted, leading to “leaky gut”, many microbial toxins and microbes might circulate systemically leading to limbs amputations as a result of such infections and reduced blood flow [3, 9]. Gut bacteria have a detrimental role in the systemic inflammation and susceptibility to nosocomial infections, to which some SCD patients are exposed as a result of frequent hospitalizations [10–14].

Cultivation-dependent techniques only give a partial picture of the gut microbiota structure and composition. With the advent of next generation high throughput technologies that are culture-independent, we have now the possibility to explore the gut microbiota structure to comprehensively identify its composition. As such, in this study, we analyzed the gut microbiome in mouse SCD models and human subjects with SCD to assess its potential associations with clinical symptoms. These results, if confirmed in a large patient cohort, should aid the design of future modulation protocols to establish a balanced gut microbiome in SCD and to determine if such interventions alter clinical outcomes.

## Materials and methods

### Patients and samples collection

All investigations in this study were conducted according to the Declaration of Helsinki. All subjects were enrolled at Howard University Hospital, Washington DC, USA under approved Institutional Review Board protocol IRB-16-MED-17. Twenty-eight adult subjects aged 22 to 57 years were recruited for this protocol from June to September 2016. All subjects provided written informed consent. These subjects consisted of 14 healthy non-SCD controls and 14 subjects with established SCD stratus. Controls and SCD groups were age and gender matched and none was on antibiotics. All 28 subjects were African Americans. Medical records were reviewed in detail to collect data regarding SCD clinical manifestations. SCD subjects were retrospectively divided into 2 groups of 7 each, based on the frequency of hospitalizations for pain in the year of recruitment. Mild group subjects (SCDM) consisted of those with less than 3 hospitalizations per year, while severe group subjects (SCDS) had more than 3 hospitalizations per year. All subjects were provided stool and saliva collection kits. The patients were given the instructions for samples’ collection. Samples were mailed on same day delivery service and stored at -80°C immediately upon receipt, as previously described [15, 16].

### DNA extraction and 16S rDNA analysis

DNA was extracted from the stool and saliva samples using QIAamp DNA Stool Mini Kit and Qiagen DNA Blood extraction kits, respectively according to the manufacturer’s instructions (Qiagen, Germantown MD, US). DNA quality was assessed using Nanodrop 2000 and gel electrophoresis. All samples yielded good quality and good amounts of DNA for bacterial community analysis. For 16S rDNA analysis, a PCR amplification was performed prior to Next Generation Sequencing (NGS) as previously described [15, 16]. Briefly, DNA extracts were amplified using primers that targeted the 16S rRNA V3-V4 gene region. These primers included adaptor sequences as well as unique 12 bp barcodes incorporated onto the reverse primer such that each sample had a unique barcode. Using approximately 100 ng of extracted DNA, the amplicons were generated with Platinum Taq polymerase (Invitrogen, CA, USA) using the following cycling conditions: 95°C for 5 min for an initial denaturing step followed by 35 cycles of: [95°C for 30s, 55°C for 30s, 72°C for 30s], followed by a final extension step of 72°C for 7min, and then stored at 4°C. PCR amplicons were purified using the QIAquick PCR purification kit (Qiagen Valencia, CA, USA), quantified, normalized, and then pooled in preparation for sequencing using an Illumina HiSeq platform according to the manufacturer’s protocol (Illumina Inc., San Diego, CA).

### NGS data processing and annotation

In the first step of data processing, the generated sequence data were deconvolved using the sample barcodes to identify sequences from each of the samples. Barcode, primer, and adaptor sequences were also trimmed as part of this step. PCR artifacts “chimeras” were identified using the ChimeraSlayer program (http://microbiomeutil.sourceforge.net; reference http://genome.cshlp.org/content/21/3/494.long) and removed prior to downstream analysis. The deconvoluted and filtered sequence data were assigned taxonomy (to the genus level) using the Ribosomal Database Project (RDP) classifier to generate a sample-genus count matrix. For Operational Taxonomic Unit (OTU) analysis, we used the Mothur software and subsequently clustered at 97% sequence identity using cd-hit to generate OTUs [16, 17]. OTU abundances were loaded into R (version 3.5.1) using the package [18]. Rarefaction curves were calculated using the R package vegan. The R package DESeQ2 [18] was used to identify taxonomic units that were significantly differentially abundant within the analyzed groups. At each taxonomic rank, we summed the read counts falling into OTUs within each taxon. These counts were then used for differential abundance analysis assuming a negative binomial distribution. The threshold for statistical significance was a false detection ratio (FDR) <0.05. For Firmicutes/Bacteroidetes ratios, we calculated the total counts in the two phyla and computed the ratio of the counts for each sample. For differential analysis of ratios, we used the Wilcoxon Rank Sum test on the ratios for each group (CTRL vs. SCD and SCDM vs. SCDS).

### SCD murine models

Two SCD mouse models were used for comparison to clinical samples. All mice were housed and treated in accordance with the Institutional Animal Care and Use study associated Control mice) and the University of Minnesota, MN (Berkeley SCD model and associated Control mice) for their respective local protocols. SCD and control mice are not littermates but derived from the same mice. AA and SS were pulled from different litters. Mice were housed with mice of the same genotype and gender. Cage, environment, and diet were all standardized among the different genotypes. Fecal material was obtained from both male and female mice; age ranged between 6 to 9 months. Stool samples were collected by our collaborators at the two respective institutions and shipped to us in dry ice, prior to them starting their own approved protocols. At the end of the protocols, the mice were euthanized using carbon dioxide based protocols.

All mice in this were obtained from the Jackson laboratory (Bar Harbor, ME). The first model utilized B6:129-*Hba*^*tm1(HBA)Tow*^ *Hbb*^*tm2(HBG1*,HBB*)Tow^ /*Hbb*^*tm3(HBG1*,*HBB)Tow*^/J strain, here referred to as the Townes (SCD) mice [19, 20]. Townes mice (homozygous HBSS, and controls, HBAA) do not express mouse hemoglobin. Homozygous Townes (hα/hα:β^S^/β^S^, HBSS) mice carry human normal α- (hα) and sickle hemoglobin beta (β^S^) genes and express over 90% of human sickle hemoglobin [21]. Control Townes (hα/hα:β^A^/β^A^, HBAA) mice carry human normal hα and β^A^ genes and express normal human hemoglobin including fetal hemoglobin [20–23]. Breeding pairs were obtained from the Jackson Laboratory (stock numbers 013071 Bar Harbor, ME) and bred in our animal facility. Genotype was determined as previously described [24]. Stool samples were collected from 7AA control and 13SS Townes mice. We also obtained stool samples from 12 homozygous HBSS Berkeley mice and 12 control HBAA mice. The Berkeley SS mouse model (Berk) was developed on a mixed genetic background with deletions of mouse α and β globins and insertion of transgenes for human α and β^S^ expressing >99% human sickle Hb; or insertion of human alpha and beta A, expressing exclusively normal human HbA (control mice). The homozygous Berk mice express >99% human sickle Hb [25–27]. Mice were bred, phenotyped for homozygosity by isoelectric focusing and housed in a regular semi-sterile facility [28].

DNA was extracted from animal stool samples as described above. After DNA quantification, PCR amplification of conserved bacterial 16S rRNA fragments was performed. PCR products were sequenced using Next Generation Sequencing (Illumina). The generated data was analyzed to define bacterial compositions and specifics within each mice group as described for clinical human samples above.

## Results

### Rarefaction curves revealed adequacy of sequencing depth for both human and mouse samples

The number of sequencing reads for each human (stool and saliva) and the two SCD mouse models are reported in S1-S4 Tables in S1 File. Rarefaction curves were established for each set of samples. For all samples, the depth of sequencing and number of reads were adequate as all curves reached OTU detection saturation (S1 Fig).

### Stool microbiome characterization in human SCD subjects and controls

The Firmicutes/Bacteroidetes ratio is an important indicator of gut microbiota homeostasis. The non-SCD control ratio for stool was 1.00004, while SCD subjects had a ratio of 0.65801 (p-value = 0.0212). Comparison of SCDM to SCDS status for SCD subjects revealed a lower SCDM ratio (0.58936), while more frequently hospitalized subjects had a ratio of 0.7341141 (p-value = 0.901). In saliva samples, Firmicutes/Bacteroidetes ratios were 1.66356 for healthy controls versus 1.42042 for SCD, and 1.09763 versus 1.75703 when comparing SCDM to SCDS subjects. None of the differences in saliva samples was significant.

**Fig 1:**
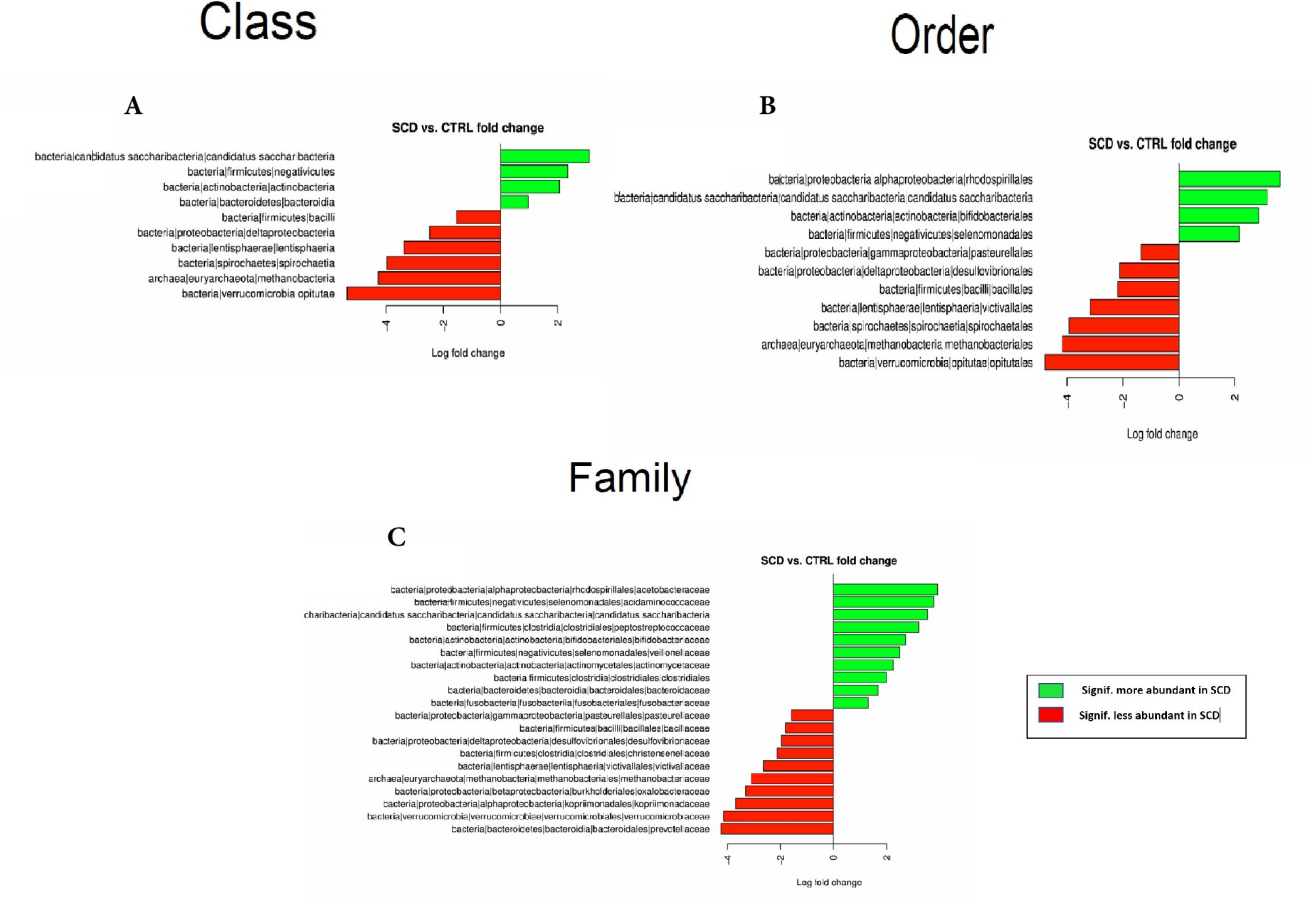
Differential bacterial Classes (A), Orders (B) and Families (C) between SCD subjects and healthy controls’ stool samples.

An abundance analysis in stool samples showed that healthy controls and SCD patients were significantly different for 10 out of 24 bacterial Classes (Fig 1A). A Deseq analysis revealed that 4 of these classes were more prevalent in SCD patients, namely: Saccharribacteria, Negativicutes, Actinobacteria and Bacteroidia. The remaining 6 significantly different Classes namely: Bacilli, Deltaproteobacteria, Entisphaeria, Spirochaetia, Methanobacteria and Opitutae were less abundant in SCD (Fig 1A).

These differences at Class level translated into differences in 11 out of 31 bacterial Orders with the following orders more abundant in SCD patients: Rhodospirillales, Candidatus Saccharibacteria, Bifidobacteriales and Selenomonadales, while Pasteurellales, Desulfovribrionales, Bacillales, Victivallales, Spirochaetales, Methanobacteriales and Opitutales were more abundant in the controls (Fig 1B).

At the family level, 20 out of 62 bacterial families were significantly different. Those more abundant in SCD patients were: Acetobacteraceae, Acidaminococcaceae, Candidatus Saccharibacteria, Peptostreptococcaceae, Bifidobacteriaceae, Veillonellaceae, Actinomycetaceae, Clostridiales, Bacteroidaceae and Fusobacteriaceae. The remaining 10 families namely: Pasteurellaceae, Bacillaceae, Desulfovibrionaceae, Christensenellaceae, Victivallaceae, Methanobacteriaceae, Oxalobacteriaceae, Kopriimonadaceae, Verrucomicrobiaceae and Prevotellaceae were significantly more abundant in the controls (Fig 1C).

**Fig 2:**
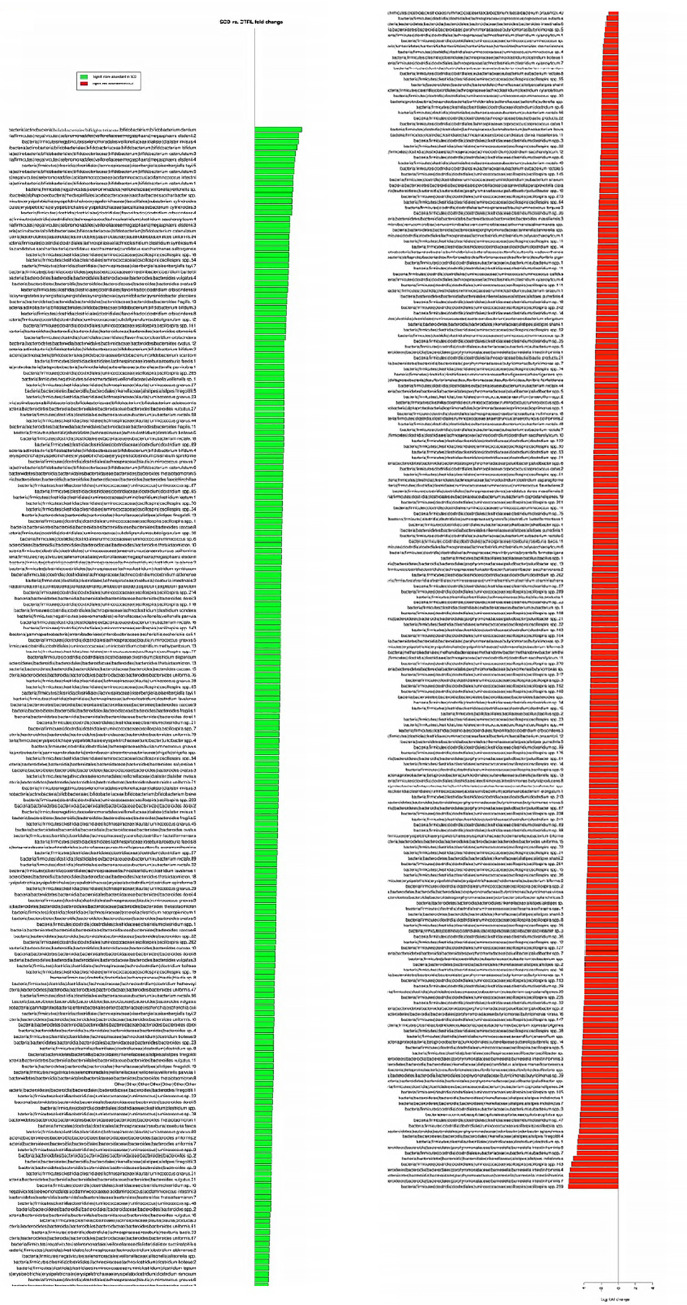
Differential OTUs between SCD subjects and healthy controls’ stool samples.

The above differences at family levels were reflected at the OTU level where a major dysbiosis was observed with 208 OTUs more abundant in SCD and 212 OTUs depleted in this group since they were significantly more abundant in non-SCD controls (Fig 2).

Sub-group analysis within the SCD revealed that there were no significant differences at Class, Order, Family or OTU levels between patients with frequent vs. less frequent hospitalizations (SCDM and SCDS groups).

### Oral microbiome analysis of human SCD versus controls

To have a good perspective on the differences observed in the stool samples, that pointed to a major dysbiosis in the gut microbiome of SCD patients, we analyzed the oral microbiome of the same patients using saliva samples.

**Fig 3:**
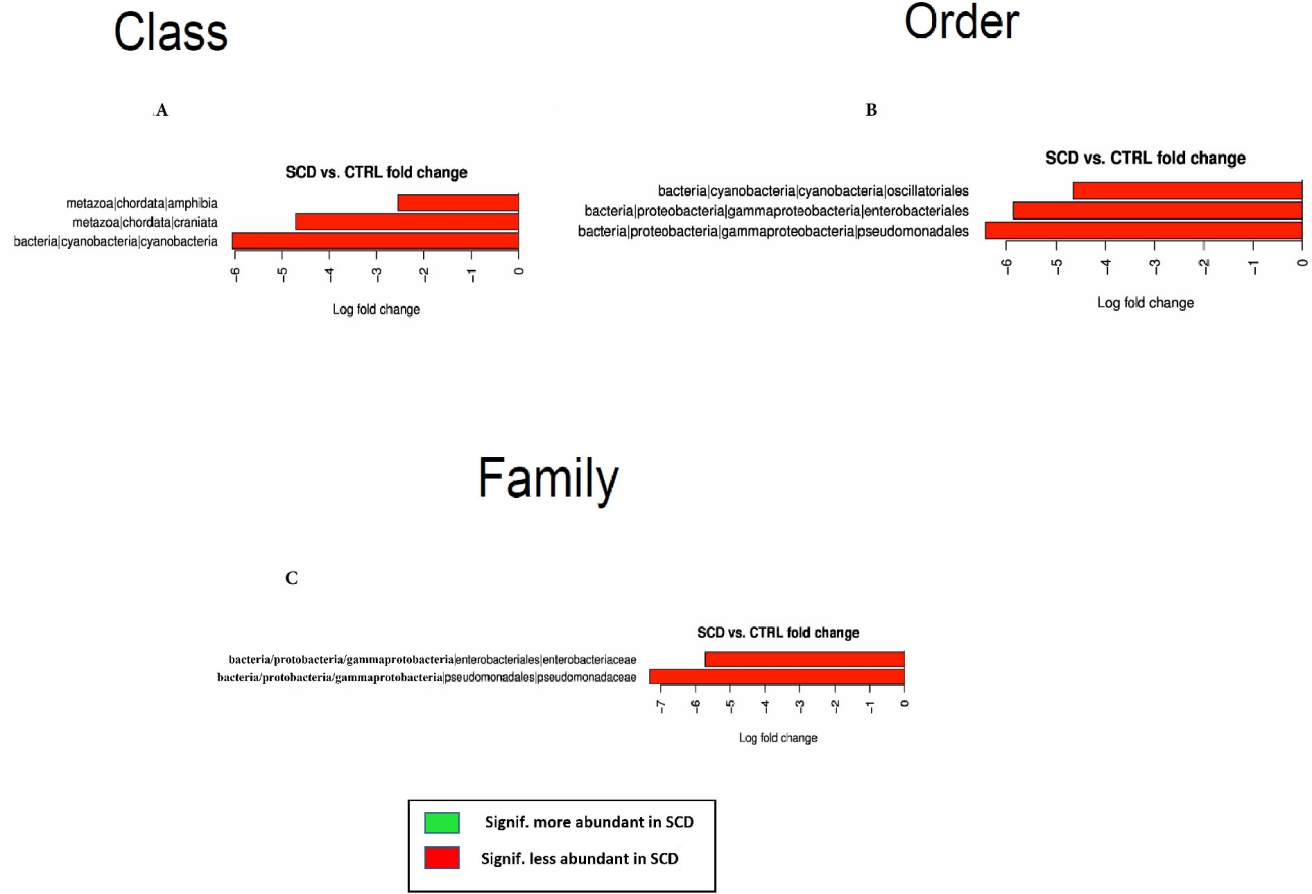
Differential bacterial Classes (A), Orders (B) and Families (C) between SCD subjects and healthy controls’ saliva samples.

While differences were noticed between oral microbiome of controls compared to SCD patients, these differences were less pronounced than those observed in the stool samples. At the Class level, Amphibia, Craniata and Cyanobacteria were significantly less abundant in SCD which translated in 3 Orders within these Classes as less abundant in SCD patients namely Oscillatoriales, Enterobacteriales, and Pseudomonadales bacteria (Fig 3A & 3B).

**Fig 4:**
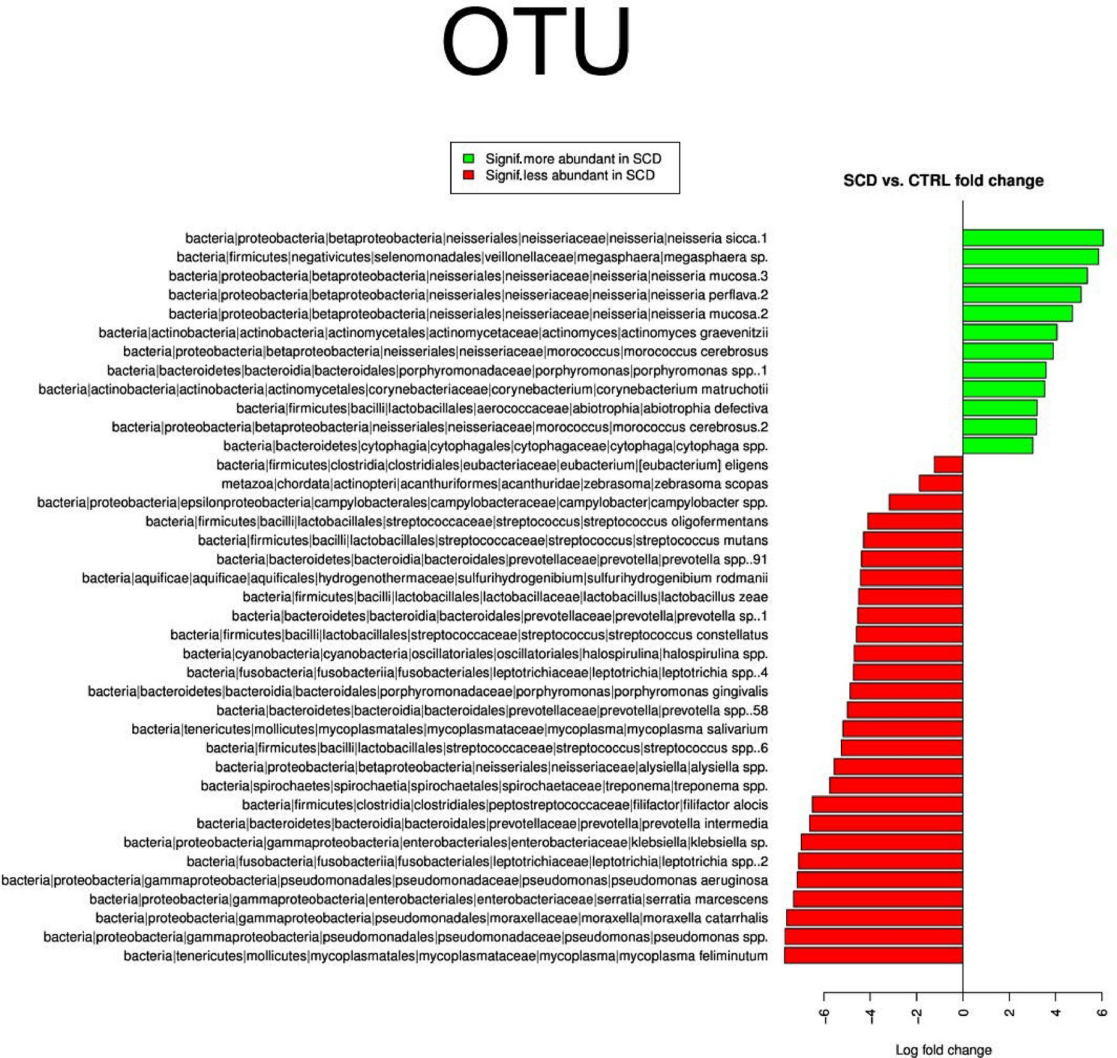
Differential OTUs between SCD subjects and healthy controls’ saliva samples.

Only 2 Families out of 100 showed significant differences and were less abundant in SCD patients saliva, Enterobacteriaceae and Pseudomonadaceae bacteria (Fig 3C). The analysis at the OTU level revealed 28 OTUs as more prevalent in healthy controls while 12 OTUs were more so in SCD patients (Fig 4).

The analysis of the oral microbiome within the SCD group revealed that there were no significant differences at any taxonomic level between SCD patients with high versus low numbers of hospitalizations (SCDM vs. SCDS).

### Microbiome analysis in SCD mouse models

To compare our findings in clinical samples and to potentially develop a model system for gut microbiome modulation studies, we characterized the gut microbiome from 2 SCD mouse models, namely the Berk and Townes mice and their associated control mice. Stool samples were obtained from 12AA and 12SS Berk mice and from 7AA and 13SS Townes SCD mice.

There were no significant differences in the gut microbiome makeup at any taxonomy level in the BERK mice, except for one Family kopriimonadaceae which was less abundant in SS BERK mice when compared to AA genotype mice. While there were differences in many OTUs abundance, none were statistically significant.

**Fig 5:**
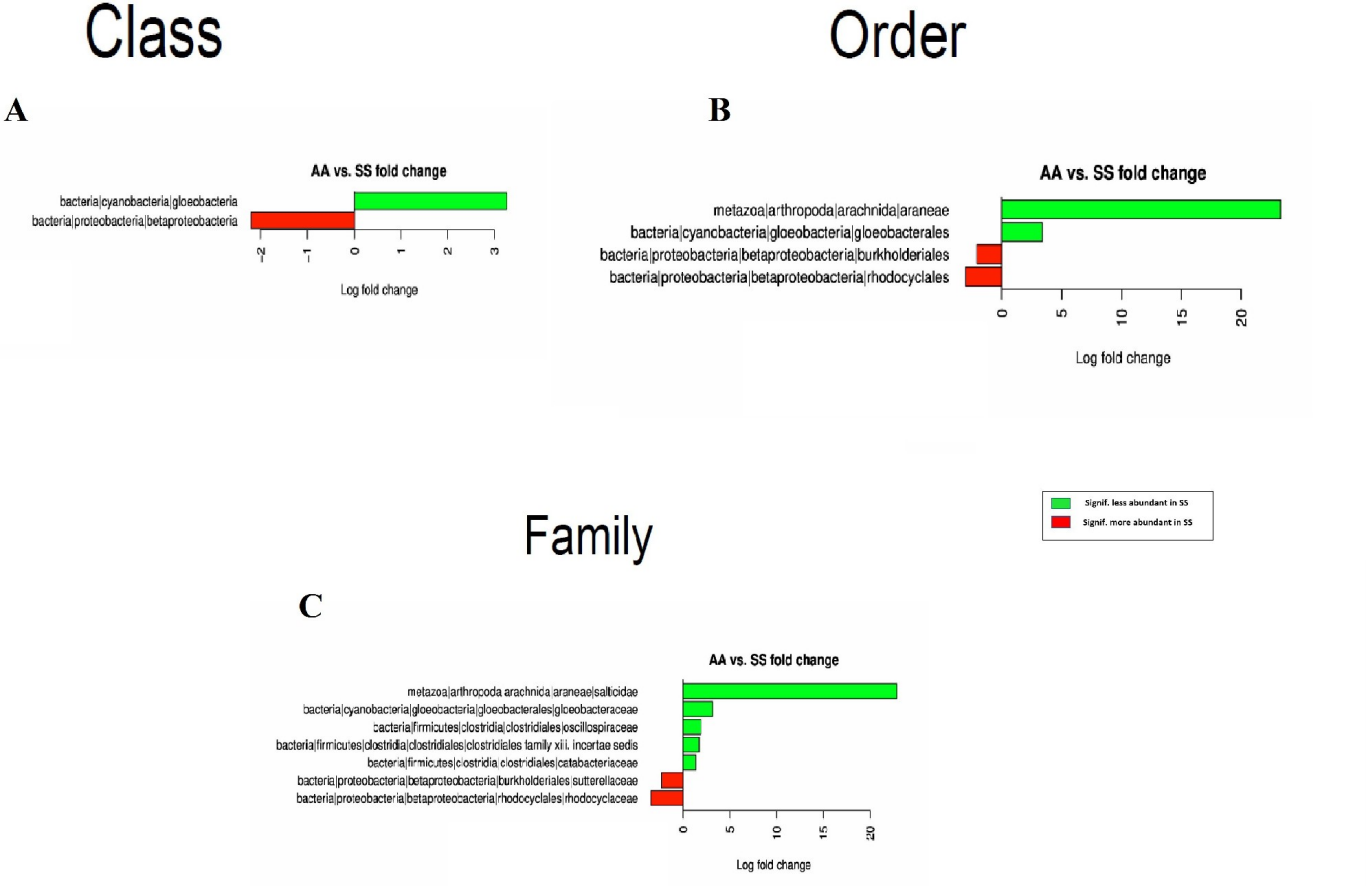
Differential bacterial Classes (A), Orders (B) and Families (C) between SS and AA Townes mice stool samples.

On the other hand, the Townes mice displayed more significant differences that are comparable to those observed in patients’ stool samples. Indeed, Cyanobacteria|gloeobacteria Class was less abundant in SS mice while bacteria from the Betaproteobacteria Class were more prevalent (Fig 5A). Four Orders within these two Classes were also significantly different with Arachnida|Araneae and Gloeobacteria|gloeobacterales as less abundant in SS mice while Betaproteobacteria|burkholderiales and Betaproteobacteria|rhodocyclales were more prevalent in SS Townes mice (Fig 5B).

**Fig 6:**
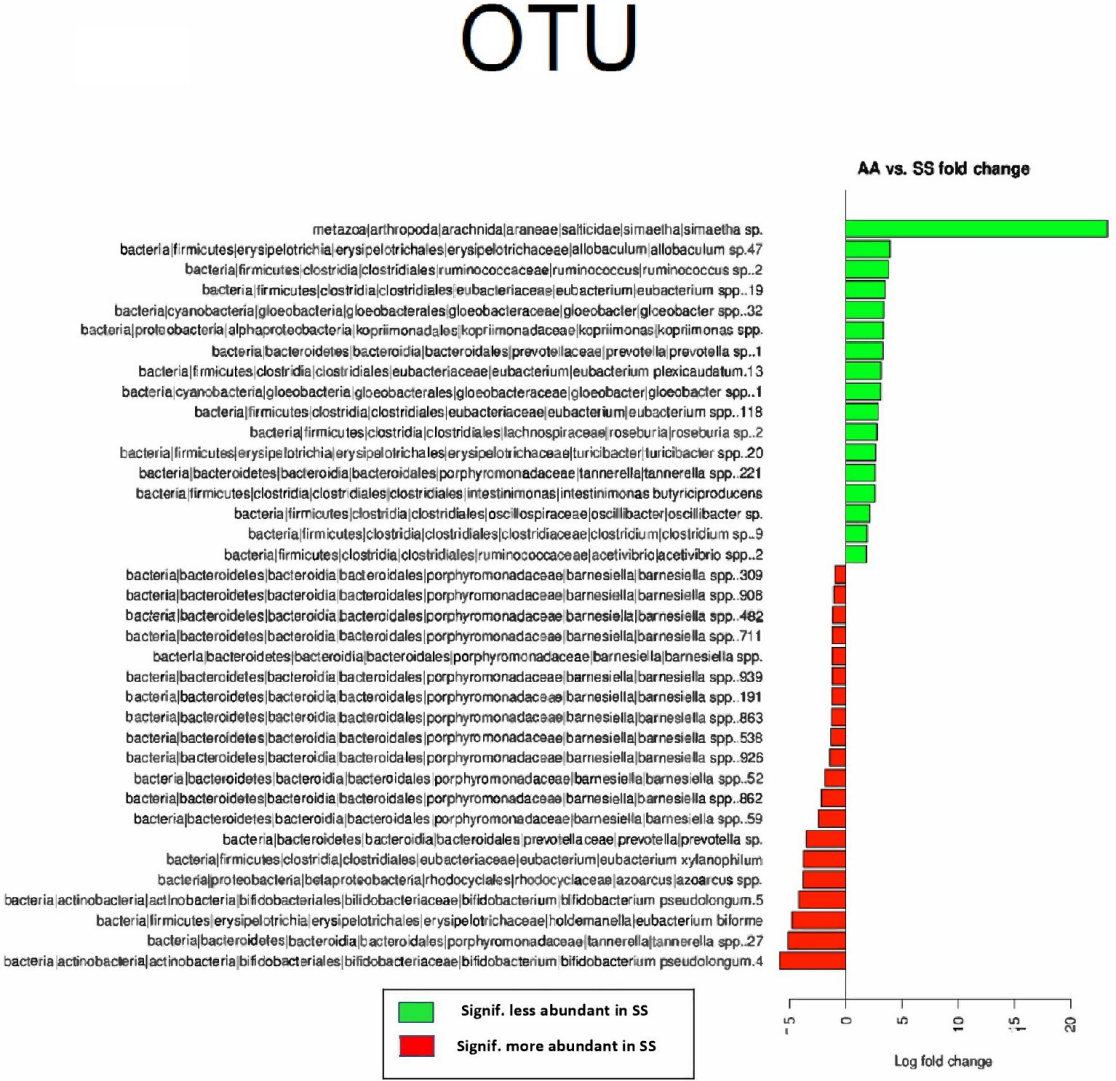
Differential OTUs between SS and AA townes mice stool samples.

At the Family level, 7 Families were statistically different with less abundant Families in the SS Townes model including Araneae|salticidae, Gloeobacterales|gloeobacteraceae Clostridiales|oscillospiraceae, Clostridiales|clostridiales family xiii. incertae sedis, Clostridiales|catabacteriaceae, while Burkholderiales|sutterellaceae and Rhodocyclales|rhodocyclaceae were more prevalent in SS Townes mice (Fig 5C). OTU analysis showed that 37 OTUs within these Families were significantly different between AA and SS Townes mice. There were 20 OTUs more abundant in SS and 17 less abundant (Fig 6).

## Discussion

We performed a comprehensive gut microbiome analysis in SCD patients that revealed a major dysbiosis, especially when compared to the saliva (oral) microbiome from the same subjects. Such SCD-associated gut microbiome dysbiosis was confirmed in a humanized SCD mouse model (Townes), but this was not observed in another commonly used mouse model (Berk).

The stool Firmicutes/Bacteroidetes ratio in SCD patients was found to be low and about half the ratio observed in the healthy controls. The Firmicutes/Bacteroidetes ratio in adults with SCD is comparable to ratios previously reported for infants and elderly subjects [29]. Since these SCD subjects are neither infants nor elderly, this ratio likely points to an aborted developmental evolution of the gut microbiome that is not observed in healthy adults. This could be the result of the ischemic conditions that prevail in SCD patients and the selective pressure exerted on the gut microbiome beginning at birth due to genetic factors like SCD and environmental factors like the use of prophylactic penicillin for the first 5 years of life [30]. It is worth noting that the Firmicutes/Bacteroidetes ratio in SCD patients and healthy controls’ saliva samples were similar and higher, respectively, than those observed in the corresponding gut microbiome ratios, especially for SCD. The evolution of the gut microbiome in SCD is likely also affected by heavy use of antibiotics at early ages in both prophylactic dosing and IV doses given in the hospital for evaluation of fevers.

Bacteria from the Bifidobacteriales Order were found to be more prevalent in SCD patients. Bacteria of this Order are some of the early colonizers of the human gut microbiome that should normally be reduced in abundance at later stages but are likely maintained as a result of the SCD gut environment. This finding has implications for future trials of probiotics in SCD. Next generation probiotics, instead of general across the board Bifidobacteria/Lactobacilli probiotics, could complement the specific needs of the SCD gut microbiota.

Further analysis of the SCD microbiome revealed that the dysbiosis is at higher taxonomic levels since comparison with controls showed significant differences in 10 bacterial Classes, 11 Orders and 20 families. At the OTU level, differences concerned 420 OTUs, of which 208 were significantly more abundant in SCD patients. To appreciate the magnitude of the observed gut microbiome dysbiosis, we conducted an oral microbiome analysis using saliva samples from the same subjects. This showed that at the class level, only 2 families had significant differences and were less abundant in SCD patients namely: Enterobacteriaceae and Pseudomonadaceae bacteria. OTU level analysis revealed 28 OTUs as more prevalent in healthy controls while 12 OTUs were more prevalent in SCD. The differences in the saliva samples highlight the significance of the gut microbiome differences between SCD and controls in this study. Furthermore, the SCD gut microbiome dysbiosis we are reporting here is more pronounced than the one reported by Lim et al. who reported differences at the genera levels but not at higher taxonomic levels between their SCD patients and AS Conrols [31]. The gut microbiome is the most consequential microbiome in the body for overall health, and our comprehensive gut microbiome analysis in SCD patients revealed a major dysbiosis which could also exacerbate the pathology of SCD.

Patients with SCD express higher levels of inflammatory cytokines including IL-6 and TNF-α, and exhibit high levels of inflammation [32]. There were many strong pro-inflammatory inducers among the bacterial families that defined the SCD gut microbiome. Anti-inflammatory drugs have been suggested as prophylaxis either together with, or instead of hydroxyurea in SCD patients [33]. Indeed, the recently approved Crizanlizumab is the first SCD therapy which directly targets inflammatory pathways and reduces the annual rate of VOCs [34, 35].

Of the predominant bacterial families in SCD, Acetobacteraceae have been involved in cases of idiopathic lymphadenitis, especially in patients with chronic granulomatous disease and as such was included in the group of bacterial pathogens [36]. Veillonellaceae were also reported to be increased in stool samples of juvenile idiopathic arthritis patients [37]. While commensal oral and gastrointestinal, and previously considered non-pathogenic, Veillonellaceae are known for their capacity to form biofilms which increase the virulence of Streptococcus strains and are increasingly associated with cases of osteomyelitis, meningitis and endocarditis [38]. Fusobacteriaceae were described in cases of periodontitis, as determinant of cardiovascular disease via translocation from the oral cavity to the systemic circulation and more recently associated with colorectal cancer susceptibility [39, 40]. While there are many pathogens within the Actinomycetaceae Family [41], Peptostreptococcaceae were generally more abundant in experimentally-induced colitis [42], another evidence of their inflammatory potential.

The cross talk between the microbiota and the immune system, and more specifically the neutrophils within innate immunity, adjusts the magnitude of neutrophil-mediated inflammation on challenge while preventing neutrophil responses against commensals and allowing opportunistic pathogens to flourish [43]. More specifically to the context of SCD, the gut microbiota is reported to affect neutrophil ageing activation processes that play major roles in vaso-occlusive crises in these patients [44]. Studies correlating gut microbiota composition and specific immune signatures are needed to further shed light on these correlations.

Our sub-group analysis within the SCD group revealed no significant differences at Class, Order, Family or OTU levels between subjects with and without frequent hospitalizations (SCDS versus SCDM groups). This finding might be the result of limited statistical power to detect an association due to small groups (n = 7 each) or it could be attributable to a true lack of correlation between gut microbiome and pain episodes. These points will be further investigated through larger trials and more precise phenotyping of hospitalizations and pain events frequencies.

There are other potential factors which could affect association analyses of gut microbiome dysbiosis including inflammatory and other host genetic factors. Tozatto-Maio et al. recently reported in an international SCD cohort from Brazil, France and Senegal that a Toll-like receptor 2 (TLR2)genetic variant modulates occurrence of bacterial infections in SCD patients [45]. As such, TLR2 variants and other genes involved in the crosstalk with the gut microbiome might be needed to shed more light on correlations between the gut microbiota dysbiosis and clinical manifestations like pain. Similarly, other factors like fetal hemoglobin levels and other host genetic factors are also associated with hospitalizations for pain.

To validate the clinical samples’ findings and to potentially have a model for gut microbiome modulation experiments, we also characterized the gut microbiome in 2 SCD mouse models, namely the Berk and Townes mice. While the Berk model showed no significant differences in the gut microbiome composition at any taxonomy level, except for the kopriimonadaceae family, Townes mice displayed significant differences comparable to the human stool microbiome in SCD. Indeed, bacteria from the Betaproteobacteria class were more prevalent in SS Townes mice. Two Orders within this Class (Betaproteobacteria|burkholderiales and Betaproteobacteria|rhodocyclales) were significantly abundant in SCD SS Townes mice, further translating to two prevalent Betaproteobacteria families (Burkholderiales|sutterellaceae and Rhodocyclales|rhodocyclaceae). The OTU analysis showed that 37 OTUs within these families were significantly different between AA and SS Townes mice, with 20 more abundant in SS and 17 more so in AA mice. These findings, while not directly comparable to clinical samples, because of intrinsic differences between human and mice gut microbiome, show that at least one SCD mouse model suggests a dysbiosis. Furthermore, Proteobacteria in general, and Betaproteobacteria are known to contain many pathogens and opportunistic pathogens that might play a similar role as the prevalent bacterial Families in SCD patients such as Acetobacteria, Fusobacteria, Actinobacyrtia and Peptostreptococaceae. Worth noting that while we carefully chose SCD patients and controls to minimize the effects of confounding factors such as age, gender and antibiotic exposure, the well-controlled mice experimental setting might suggest that the dysbiosis observed in the Twones mice is solely and predominantly driven by conditions associated with the SCD phenotype. These findings support further exploration of this mouse model for gut microbiome modulation effect studies in SCD. These studies might also use germ free Townes mice that can receive fecal matter transplants from healthy humans and those with SCD to determine if these mice would respond and reshape the transplanted gut microbiota. These experiments might also challenge the transplanted mice to assess their pathogen colonization resistance and associated immune responses.

In conclusion, we have generated a descriptive analysis of the gut microbiome in adults with SCD which suggests a major dysbiosis at higher taxonomy levels. Our data provide a foundation to launch the application of next generation probiotics specifically designed for SCD patients to potentially reduce gut microbiota-driven inflammation, which may ultimately mitigate the severity of VOC and other end organ damage.

## Supporting information

S1 Fig Rarefaction curves were established for each set of samples. For all samples, the depth of sequencing and number of reads were adequate as all curves reached OTU detection saturation.
(TIF)

S1 File Number of sequencing reads for human specimens (stool and saliva) and the two SCD mouse models stool samples. (PDF)

S2 File Operational taxonomic units’ differences between SCD patients and healthy controls. (DOCX)

## Abbreviations

SCD: Sickle cell disease
VOC: Vaso-Occlusive Crises
OTU: Operational Taxonomic Unit

## References

[1] Kato G.J., et al., Sickle cell disease. Nat Rev Dis Primers, 2018. 4: p. 18010. doi: 10.1038/nrdp.2018.10 29542687

[2] Amid A. and Odame I., Improving outcomes in children with sickle cell disease: treatment considerations and strategies. Paediatr Drugs, 2014. 16(4): p. 255–66. doi: 10.1007/s40272-014-0074-4 24797542

[3] Maximo C., et al., Amputations in Sickle Cell Disease: Case Series and Literature Review. Hemoglobin, 2016. 40(3): p. 150–5. doi: 10.3109/03630269.2016.1167736 27117565

[4] Shreiner A.B., Kao J.Y., and Young V.B., The gut microbiome in health and in disease. Curr Opin Gastroenterol, 2015. 31(1): p. 69–75. doi: 10.1097/MOG.0000000000000139 25394236 PMC4290017

[5] Dinan T.G. and Cryan J.F., The Microbiome-Gut-Brain Axis in Health and Disease. Gastroenterol Clin North Am, 2017. 46(1): p. 77–89. doi: 10.1016/j.gtc.2016.09.007 28164854

[6] Ehrlich S.D., The human gut microbiome impacts health and disease. C R Biol, 2016. 339(7–8): p. 319–23. doi: 10.1016/j.crvi.2016.04.008 27236827

[7] Bull M.J. and Plummer N.T., Part 1: The Human Gut Microbiome in Health and Disease. Integr Med (Encinitas), 2014. 13(6): p. 17–22. 26770121 PMC4566439

[8] Wu G.D., The Gut Microbiome, Its Metabolome, and Their Relationship to Health and Disease. Nestle Nutr Inst Workshop Ser, 2016. 84: p. 103–10. doi: 10.1159/000436993 26764479

[9] Gouveia C., et al., Osteoarticular infections in paediatric sickle cell disease: in the era of multidrugresistant bacteria. Br J Haematol, 2020. 189(4): p. e147–e150. doi: 10.1111/bjh.16568 32150291

[10] Bansil N.H., et al., Incidence of serious bacterial infections in febrile children with sickle cell disease. Clin Pediatr (Phila), 2013. 52(7): p. 661–6. doi: 10.1177/0009922813488645 23661790

[11] Alima Yanda A.N., et al., Burden and spectrum of bacterial infections among sickle cell disease children living in Cameroon. BMC Infect Dis, 2017. 17(1): p. 211. doi: 10.1186/s12879-017-2317-9 28298206 PMC5353947

[12] Obaro S.K. and Iroh Tam P.Y., Preventing Infections in Sickle Cell Disease: The Unfinished Business. Pediatr Blood Cancer, 2016. 63(5): p. 781–5. doi: 10.1002/pbc.25911 26840500

[13] Odey F., Okomo U., and Oyo-Ita A., Vaccines for preventing invasive salmonella infections in people with sickle cell disease. Cochrane Database Syst Rev, 2015(6): p. CD006975. doi: 10.1002/14651858.CD006975.pub3 26043710

[14] Kondani D.A., et al., Prevalence of sickle cell disease in a pediatric population suffering from severe infections: a Congolese experience. Hemoglobin, 2014. 38(4): p. 225–9. doi: 10.3109/03630269.2014.917658 25023084

[15] Brim H., et al., A Microbiomic Analysis in African Americans with Colonic Lesions Reveals Streptococcus sp.VT162 as a Marker of Neoplastic Transformation. Genes (Basel), 2017. 8(11). doi: 10.3390/genes8110314 29120399 PMC5704227

[16] Brim H., et al., Microbiome analysis of stool samples from African Americans with colon polyps. PLoS One, 2013. 8(12): p. e81352. doi: 10.1371/journal.pone.0081352 24376500 PMC3869648

[17] Love M.I., Huber W., and Anders S., Moderated estimation of fold change and dispersion for RNA-seq data with DESeq2. Genome Biol, 2014. 15(12): p. 550. doi: 10.1186/s13059-014-0550-8 25516281 PMC4302049

[18] McMurdie P.J. and Holmes S., phyloseq: an R package for reproducible interactive analysis and graphics of microbiome census data. PLoS One, 2013. 8(4): p. e61217. doi: 10.1371/journal.pone.0061217 23630581 PMC3632530

[19] Ryan T.M., Ciavatta D.J., and Townes T.M., Knockout-transgenic mouse model of sickle cell disease. Science, 1997. 278(5339): p. 873–6. doi: 10.1126/science.278.5339.873 9346487

[20] Wu L.C., et al., Correction of sickle cell disease by homologous recombination in embryonic stem cells. Blood, 2006. 108(4): p. 1183–8. doi: 10.1182/blood-2006-02-004812 16638928 PMC1895869

[21] Khaibullina A., et al., Rapamycin increases fetal hemoglobin and ameliorates the nociception phenotype in sickle cell mice. Blood Cells Mol Dis, 2015. 55(4): p. 363–72. doi: 10.1016/j.bcmd.2015.08.001 26460261

[22] Almeida L.E., et al., Validation of a method to directly and specifically measure nitrite in biological matrices. Nitric Oxide, 2015. 45: p. 54–64. doi: 10.1016/j.niox.2014.10.008 25445633

[23] Kenyon N., et al., Sickle cell disease in mice is associated with sensitization of sensory nerve fibers. Exp Biol Med (Maywood), 2015. 240(1): p. 87–98. doi: 10.1177/1535370214544275 25070860 PMC4935179

[24] Almeida L.E., et al., Immunohistochemical expression of matrix metalloprotease-2 and matrix metalloprotease-9 in the disks of patients with temporomandibular joint dysfunction. J Oral Pathol Med, 2015. 44(1): p. 75–9. doi: 10.1111/jop.12213 25065390

[25] Manci E.A., et al., Pathology of Berkeley sickle cell mice: similarities and differences with human sickle cell disease. Blood, 2006. 107(4): p. 1651–8. doi: 10.1182/blood-2005-07-2839 16166585 PMC1895417

[26] Paszty C., et al., Transgenic knockout mice with exclusively human sickle hemoglobin and sickle cell disease. Science, 1997. 278(5339): p. 876–8. doi: 10.1126/science.278.5339.876 9346488

[27] Paszty C., Transgenic and gene knock-out mouse models of sickle cell anemia and the thalassemias. Curr Opin Hematol, 1997. 4(2): p. 88–93. doi: 10.1097/00062752-199704020-00003 9107524

[28] Sagi V., et al., Mouse Models of Pain in Sickle Cell Disease. Curr Protoc Neurosci, 2018. 85(1): p. e54. doi: 10.1002/cpns.54 30265442

[29] Mariat D., et al., The Firmicutes/Bacteroidetes ratio of the human microbiota changes with age. BMC Microbiol, 2009. 9: p. 123. doi: 10.1186/1471-2180-9-123 19508720 PMC2702274

[30] Gaston M.H., et al., Prophylaxis with oral penicillin in children with sickle cell anemia. A randomized trial. N Engl J Med, 1986. 314(25): p. 1593–9. doi: 10.1056/NEJM198606193142501 3086721

[31] Lim S.H., et al., Intestinal microbiome analysis revealed dysbiosis in sickle cell disease. Am J Hematol, 2018. 93(4): p. E91–E93. doi: 10.1002/ajh.25019 29274089

[32] Niu X., et al., Angiogenic and inflammatory markers of cardiopulmonary changes in children and adolescents with sickle cell disease. PLoS One, 2009. 4(11): p. e7956. doi: 10.1371/journal.pone.0007956 19956689 PMC2776981

[33] Conran N. and Belcher J.D., Inflammation in sickle cell disease. Clin Hemorheol Microcirc, 2018. 68(2–3): p. 263–299. doi: 10.3233/CH-189012 29614637 PMC6314308

[34] Ataga K.I., Kutlar A., and Kanter J., Crizanlizumab in Sickle Cell Disease. N Engl J Med, 2017. 376(18): p. 1796. doi: 10.1056/NEJMc1703162 28467874

[35] Ataga K.I., et al., Crizanlizumab for the Prevention of Pain Crises in Sickle Cell Disease. N Engl J Med, 2017. 376(5): p. 429–439. doi: 10.1056/NEJMoa1611770 27959701 PMC5481200

[36] Fredricks D. and Ramakrishnan L., The Acetobacteraceae: extending the spectrum of human pathogens. PLoS Pathog, 2006. 2(4): p. e36. doi: 10.1371/journal.ppat.0020036 16652172 PMC1447671

[37] Di Paola M., et al., Alteration of Fecal Microbiota Profiles in Juvenile Idiopathic Arthritis. Associations with HLA-B27 Allele and Disease Status. Front Microbiol, 2016. 7: p. 1703. doi: 10.3389/fmicb.2016.01703 27833598 PMC5080347

[38] Hirai J., et al., Osteomyelitis caused by Veillonella species: Case report and review of the literature. J Infect Chemother, 2016. 22(6): p. 417–20. doi: 10.1016/j.jiac.2015.12.015 26857179

[39] Kelly D., Yang L., and Pei Z., Gut Microbiota, Fusobacteria, and Colorectal Cancer. Diseases, 2018. 6(4). 10.3390/diseases6040109 PMC6313651 30544946

[40] Sears C.L., The who, where and how of fusobacteria and colon cancer. Elife, 2018. 7. 10.7554/eLife.28434 PMC5849411 29533185

[41] Kononen E. and Wade W.G., Actinomyces and related organisms in human infections. Clin Microbiol Rev, 2015. 28(2): p. 419–42. doi: 10.1128/CMR.00100-14 25788515 PMC4402957

[42] Chen W., et al., Taraxacum officinale extract ameliorates dextran sodium sulphate-induced colitis by regulating fatty acid degradation and microbial dysbiosis. J Cell Mol Med, 2019. 23(12): p. 8161–8172. doi: 10.1111/jcmm.14686 31565850 PMC6850927

[43] Zhang D. and Frenette P.S., Cross talk between neutrophils and the microbiota. Blood, 2019. 133(20): p. 2168–2177. doi: 10.1182/blood-2018-11-844555 30898860 PMC6524562

[44] Dutta D., et al., Intestinal pathophysiological and microbial changes in sickle cell disease: Potential targets for therapeutic intervention. Br J Haematol, 2020. 188(4): p. 488–493. doi: 10.1111/bjh.16273 31693163

[45] Tozatto-Maio K., et al., A Toll-like receptor 2 genetic variant modulates occurrence of bacterial infections in patients with sickle cell disease. Br J Haematol, 2019. 185(5): p. 918–924. doi: 10.1111/bjh.15875 30908604

