# Supplementary figures and images for: The gut microbiome in sickle cell disease: Characterization and potential implications
Source: PLoS One. 2021 Aug 25;16(8):e0255956. doi: 10.1371/journal.pone.0255956 (PMC8386827; doi:10.1371/journal.pone.0255956)

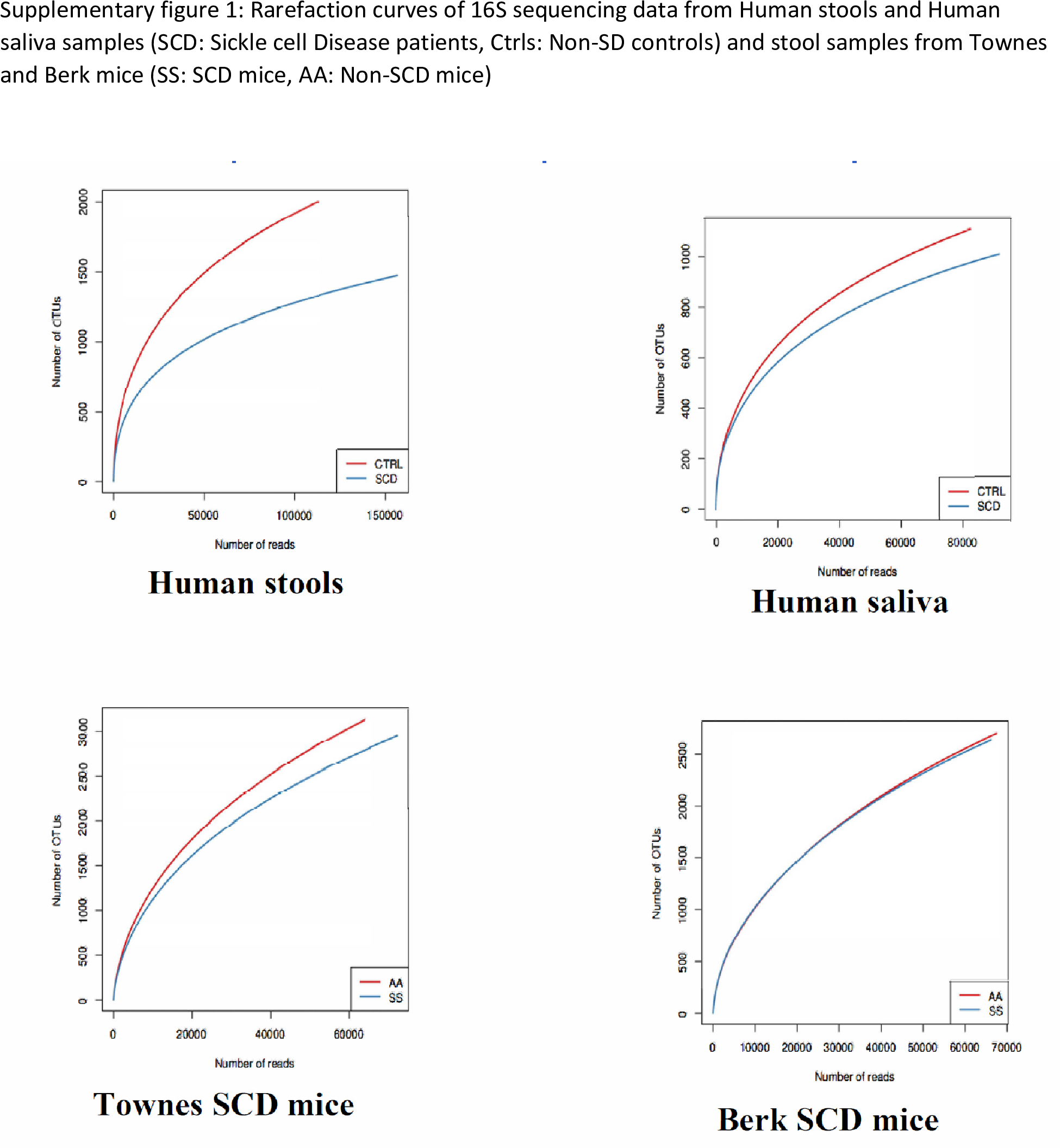

Supplement: S1 Fig — For all samples, the depth of sequencing and number of reads were adequate as all curves reached OTU detection saturation. (TIF) [file pone.0255956.s001.tif]
