# Supplementary material for: The gut microbiome in sickle cell disease: Characterization and potential implications
Source: PLoS One. 2021 Aug 25;16(8):e0255956. doi: 10.1371/journal.pone.0255956 (PMC8386827; doi:10.1371/journal.pone.0255956)
Supplement: S1 File — (PDF) [file pone.0255956.s002.pdf]

S1 File

The Gut Microbiome in Sickle Cell Disease: Characterization and Potential Implications

Hassan Brim1, James Taylor, Muneer Abbas, Kimberly Vilmenay, Mohammad Daremipouran, Sudhir Varma, Edward Lee, Betty Pace, Waogwende L. Song-Naba, Kalpna Gupta, Sergei Nekhai, Patricia O'Neil, and Hassan Ashktorab

Supplementary Tables

S1 Table: Human stool samples' read counts (Ctrl. Non-SCD Controls, SCDM: SCD patients with <3 hospitalizations/year, SCDS: SCD patients with> 3 hospitalizations/year)

Sample	Disease	Total reads	Mapped to OTUs	Mapped to chimeras	Unmapped	Total OTUs
CTRL1	CTRL	155968	123288	20350	12330	1645
CTRL2	CTRL	140574	113014	16340	11220	1365
CTRL3	CTRL	132993	103906	18173	10914	1189
CTRL4	CTRL	150536	121296	18354	10886	1109
CTRL5	CTRL	92144	73746	10603	7795	1460
CTRL6	CTRL	124789	103758	12137	8894	1543
CTRL7	CTRL	151155	127792	13257	10106	1383
CTRL8	CTRL	137592	109973	16661	10958	1621
CTRL9	CTRL	120244	94672	15560	10012	1632
CTRL10	CTRL	133844	103985	17938	11921	1238
CTRL11	CTRL	134060	106722	17508	9830	1472
CTRL12	CTRL	148172	129027	10001	9144	1271
CTRL13	CTRL	153020	126743	14968	11309	1434
CTRL14	CTRL	179427	145177	19861	14389	1372
SCDM1	SCDM	179207	144409	20932	13866	1033
SCDM2	SCDM	158670	130455	14627	13588	1225
SCDM3	SCDM	242597	201024	27317	14256	864
SCDM4	SCDM	170827	138205	21258	11364	1131
SCDM5	SCDM	151714	119406	19420	12888	1339
SCDM6	SCDM	212330	176836	20887	14607	938
SCDM7	SCDM	175172	149872	14488	10812	1298
SCDS1	SCDS	179983	150258	16926	12799	969
SCDS2	SCDS	165758	126589	26766	12403	1016
SCDS3	SCDS	198667	156083	25796	16788	1007
SCDS4	SCDS	214492	178392	22807	13293	1119
SCDS5	SCDS	221348	193045	16280	12023	1085
SCDS6	SCDS	213195	175869	21925	15401	877
SCDS7	SCDS	189684	154199	21989	13496	1008

S2 Table: Human saliva samples' read counts (Ctrl. Non-SCD Controls, SCDM: SCD patients with <3 hospitalizations/year, SCDS: SCD patients with> 3 hospitalizations/year)

Sample	Disease	Total reads	Mapped to OTUs	Mapped to chimeras	Unmapped	Total OTUs
CTRL1	CTRL	155968	123288	20350	12330	1645
CTRL2	CTRL	140574	113014	16340	11220	1365
CTRL3	CTRL	132993	103906	18173	10914	1189
CTRL4	CTRL	150536	121296	18354	10886	1109
CTRL5	CTRL	92144	73746	10603	7795	1460
CTRL6	CTRL	124789	103758	12137	8894	1543
CTRL7	CTRL	151155	127792	13257	10106	1383
CTRL8	CTRL	137592	109973	16661	10958	1621
CTRL9	CTRL	120244	94672	15560	10012	1632
CTRL10	CTRL	133844	103985	17938	11921	1238
CTRL11	CTRL	134060	106722	17508	9830	1472
CTRL12	CTRL	148172	129027	10001	9144	1271
CTRL13	CTRL	153020	126743	14968	11309	1434
CTRL14	CTRL	179427	145177	19861	14389	1372
SCDM1	SCDM	179207	144409	20932	13866	1033
SCDM2	SCDM	158670	130455	14627	13588	1225
SCDM3	SCDM	242597	201024	27317	14256	864
SCDM4	SCDM	170827	138205	21258	11364	1131
SCDM5	SCDM	151714	119406	19420	12888	1339
SCDM6	SCDM	212330	176836	20887	14607	938
SCDM7	SCDM	175172	149872	14488	10812	1298
SCDS1	SCDS	179983	150258	16926	12799	969
SCDS2	SCDS	165758	126589	26766	12403	1016
SCDS3	SCDS	198667	156083	25796	16788	1007
SCDS4	SCDS	214492	178392	22807	13293	1119
SCDS5	SCDS	221348	193045	16280	12023	1085
SCDS6	SCDS	213195	175869	21925	15401	877
SCDS7	SCDS	189684	154199	21989	13496	1008

S3 Table: Townes mice stool samples' read counts (AA: Non-SCD mice, SS: SCD mice)

Sample	Genotype	Total reads	Mapped to OTUs	Mapped to chimeras	Unmapped	Total OTUs
P3932AA	AA	77499	66396	2374	8729	3059
P3933AA	AA	69366	60556	2336	6474	2669
P3937AA	AA	69640	60821	1869	6950	2186
P3946AA	AA	79600	69441	1991	8168	2786
P3949AA	AA	67964	59557	1528	6879	2872
P3950AA	AA	76722	67006	1837	7879	2999
P4951AA	AA	75327	66509	1788	7030	2710
P3914SS	SS	72236	63119	1796	7321	2563
P3911SS	SS	84463	74666	1683	8114	2358
P3998SS	SS	81612	71955	1652	8005	2666
P3928SS	SS	86525	74965	3733	7827	2364
P4248SS	SS	73138	63938	1665	7535	2788
P3930SS	SS	83238	73681	1340	8217	2523
P3929SS	SS	89258	79559	2029	7670	2503
P4969SS	SS	107594	94022	4122	9450	2496
P3936SS	SS	73490	64702	1846	6942	2442
P3927SS	SS	83455	72231	2445	8779	2223
P4968SS	SS	80278	71676	1853	6749	2303
P3947SS	SS	69247	61999	713	6535	2091
P3913SS	SS	88887	78455	978	9454	2687

S4 Table: Berk mice stool samples' read counts (AA: Non-SCD mice, SS: SCD mice)

Sample	Genotype	Total reads	Mapped to OTUs	Mapped to chimeras	Unmapped	Total OTUs
G9962SS	SS	83366	74676	2142	6548	2217
G9951SS	SS	69167	61524	1767	5876	2204
G9952SS	SS	59608	51559	2151	5898	2460
G9969SS	SS	79215	69894	2230	7091	2598
G9960SS	SS	75250	65677	2130	7443	2607
G9961SS	SS	76399	68495	1724	6180	2375
G9932SS	SS	82270	71066	4761	6443	2043
G9936SS	SS	69490	61390	2259	5841	2396
G9942SS	SS	83848	73305	3512	7031	2240
G9943SS	SS	77443	69317	1966	6160	2294
G9974SS	SS	71617	61963	2322	7332	2528
G9975SS	SS	82096	72345	2767	6984	1976
G1028AA	AA	72321	63675	2924	5722	1911
G10029AA	AA	99296	86451	4523	8322	2263
G10030AA	AA	76518	66368	2885	7265	2723
G10031AA	AA	77078	68360	1938	6780	2615
G023AA	AA	69035	60445	1958	6632	2559
G024AA	AA	84790	76220	2106	6464	2405
G022AA	AA	87793	76011	4899	6883	2064
G025AA	AA	78933	69073	2740	7120	2486
G026AA	AA	90151	78797	3638	7716	2330
G017AA	AA	73549	65939	1686	5924	2133
G018AA	AA	41125	35116	1288	4721	1886
G019AA	AA	79133	70620	2411	6102	1822
