# Supplementary material for: The gut microbiome in sickle cell disease: Characterization and potential implications
Source: PLoS One. 2021 Aug 25;16(8):e0255956. doi: 10.1371/journal.pone.0255956 (PMC8386827; doi:10.1371/journal.pone.0255956)
Supplement: S2 File — (DOCX) [file pone.0255956.s003.docx]

S2 File: Operational Taxonomic Units differeneces between SCD patients and Healthy Controls (CTRL).

| **Taxon** | **Average count** | **Log2 fold change (SCD/CTRL)** | **P-value** | **FDR (adjusted P-value)** |
| --- | --- | --- | --- | --- |
| bacteria\|bacteroidetes\|bacteroidia\|bacteroidales\|bacteroidaceae\|bacteroides\|bacteroides vulgatus | 22557.19 | 2.12 | 0.000 | 0.001 |
| bacteria\|bacteroidetes\|bacteroidia\|bacteroidales\|rikenellaceae\|alistipes\|alistipes finegoldii | 2430.25 | 2.07 | 0.011 | 0.041 |
| bacteria\|firmicutes\|negativicutes\|selenomonadales\|veillonellaceae\|dialister\|dialister invisus | 4854.92 | 2.61 | 0.005 | 0.023 |
| bacteria\|proteobacteria\|betaproteobacteria\|burkholderiales\|sutterellaceae\|parasutterella\|parasutterella excrementihominis | 1277.33 | 2.47 | 0.002 | 0.013 |
| bacteria\|verrucomicrobia\|verrucomicrobiae\|verrucomicrobiales\|verrucomicrobiaceae\|akkermansia\|akkermansia spp. | 134.62 | -2.31 | 0.001 | 0.004 |
| bacteria\|proteobacteria\|gammaproteobacteria\|enterobacteriales\|enterobacteriaceae\|escherichia\|escherichia coli | 2229.31 | 2.12 | 0.011 | 0.040 |
| bacteria\|bacteroidetes\|bacteroidia\|bacteroidales\|bacteroidaceae\|bacteroides\|bacteroides thetaiotaomicron | 999.15 | 2.31 | 0.000 | 0.001 |
| bacteria\|bacteroidetes\|bacteroidia\|bacteroidales\|bacteroidaceae\|bacteroides\|bacteroides ovatus | 4699.40 | 2.52 | 0.000 | 0.001 |
| bacteria\|firmicutes\|clostridia\|clostridiales\|ruminococcaceae\|oscillospira\|oscillospira spp. | 2560.95 | -4.50 | 0.000 | 0.000 |
| bacteria\|actinobacteria\|actinobacteria\|bifidobacteriales\|bifidobacteriaceae\|bifidobacterium\|bifidobacterium adolescentis | 3514.79 | 3.19 | 0.001 | 0.005 |
| bacteria\|firmicutes\|negativicutes\|selenomonadales\|acidaminococcaceae\|acidaminococcus\|acidaminococcus intestini | 441.02 | 4.18 | 0.000 | 0.000 |
| bacteria\|firmicutes\|clostridia\|clostridiales\|lachnospiraceae\|blautia\|ruminococcus gnavus | 720.57 | 2.63 | 0.000 | 0.000 |
| bacteria\|bacteroidetes\|bacteroidia\|bacteroidales\|porphyromonadaceae\|tannerella\|tannerella spp. | 141.12 | -2.31 | 0.000 | 0.002 |
| bacteria\|firmicutes\|clostridia\|clostridiales\|clostridiaceae\|clostridium\|clostridium spp. | 100.24 | 1.95 | 0.000 | 0.000 |
| bacteria\|fusobacteria\|fusobacteriia\|fusobacteriales\|fusobacteriaceae\|fusobacterium\|fusobacterium mortiferum | 19.94 | 0.77 | 0.011 | 0.040 |
| bacteria\|bacteroidetes\|bacteroidia\|bacteroidales\|porphyromonadaceae\|barnesiella\|barnesiella intestinihominis | 635.54 | -5.70 | 0.000 | 0.000 |
| bacteria\|firmicutes\|erysipelotrichia\|erysipelotrichales\|erysipelotrichaceae\|erysipelatoclostridium\|clostridium spiroforme | 195.44 | 3.11 | 0.000 | 0.000 |
| bacteria\|firmicutes\|erysipelotrichia\|erysipelotrichales\|erysipelotrichaceae\|holdemanella\|eubacterium biforme | 77.56 | -3.51 | 0.000 | 0.000 |
| bacteria\|actinobacteria\|actinobacteria\|bifidobacteriales\|bifidobacteriaceae\|bifidobacterium\|bifidobacterium breve | 45.27 | 2.58 | 0.000 | 0.000 |
| bacteria\|proteobacteria\|betaproteobacteria\|burkholderiales\|sutterellaceae\|sutterella\|sutterella wadsworthensis | 227.44 | -2.44 | 0.000 | 0.002 |
| bacteria\|firmicutes\|clostridia\|clostridiales\|ruminococcaceae\|oscillospira\|oscillospira spp..1 | 668.68 | 3.00 | 0.000 | 0.000 |
| bacteria\|proteobacteria\|deltaproteobacteria\|desulfovibrionales\|desulfovibrionaceae\|desulfovibrio\|desulfovibrio spp. | 265.48 | -4.26 | 0.000 | 0.000 |
| bacteria\|firmicutes\|clostridia\|clostridiales\|lachnospiraceae\|lachnoclostridium\|clostridium bolteae | 495.24 | 2.20 | 0.000 | 0.001 |
| bacteria\|firmicutes\|clostridia\|clostridiales\|ruminococcaceae\|oscillospira\|oscillospira spp..2 | 502.16 | -3.67 | 0.000 | 0.000 |
| bacteria\|firmicutes\|negativicutes\|selenomonadales\|veillonellaceae\|megasphaera\|megasphaera elsdenii | 66.62 | 2.94 | 0.000 | 0.000 |
| archaea\|euryarchaeota\|methanobacteria\|methanobacteriales\|methanobacteriaceae\|methanobrevibacter\|methanobrevibacter smithii | 15.16 | -3.16 | 0.000 | 0.002 |
| bacteria\|proteobacteria\|betaproteobacteria\|burkholderiales\|sutterellaceae\|sutterella\|sutterella spp. | 42.14 | -1.94 | 0.003 | 0.017 |
| bacteria\|firmicutes\|negativicutes\|selenomonadales\|acidaminococcaceae\|acidaminococcus\|acidaminococcus fermentans | 19.02 | 1.44 | 0.014 | 0.049 |
| bacteria\|firmicutes\|clostridia\|clostridiales\|ruminococcaceae\|ruminococcus\|ruminococcus sp. | 135.93 | -1.68 | 0.003 | 0.015 |
| bacteria\|firmicutes\|clostridia\|clostridiales\|clostridiaceae\|clostridium\|clostridium sp..1 | 377.40 | -4.72 | 0.000 | 0.000 |
| bacteria\|firmicutes\|negativicutes\|selenomonadales\|veillonellaceae\|veillonella\|veillonella parvula | 72.89 | 2.82 | 0.000 | 0.000 |
| bacteria\|firmicutes\|clostridia\|clostridiales\|eubacteriaceae\|eubacterium\|eubacterium coprostanoligenes | 346.15 | -4.08 | 0.000 | 0.000 |
| bacteria\|firmicutes\|clostridia\|clostridiales\|ruminococcaceae\|ruminiclostridium\|clostridium leptum | 118.43 | 1.56 | 0.012 | 0.045 |
| bacteria\|firmicutes\|erysipelotrichia\|erysipelotrichales\|erysipelotrichaceae\|erysipelatoclostridium\|clostridium ramosum | 72.42 | 1.52 | 0.005 | 0.024 |
| bacteria\|firmicutes\|clostridia\|clostridiales\|clostridiaceae\|clostridium\|clostridium spp..1 | 74.41 | 2.30 | 0.000 | 0.001 |
| bacteria\|firmicutes\|clostridia\|clostridiales\|ruminococcaceae\|acetanaerobacterium\|acetanaerobacterium elongatum | 61.05 | -2.57 | 0.000 | 0.001 |
| bacteria\|firmicutes\|clostridia\|clostridiales\|lachnospiraceae\|lachnoclostridium\|clostridium xylanolyticum | 255.50 | -1.89 | 0.003 | 0.014 |
| bacteria\|actinobacteria\|actinobacteria\|bifidobacteriales\|bifidobacteriaceae\|bifidobacterium\|bifidobacterium bifidum | 418.65 | 4.61 | 0.000 | 0.000 |
| bacteria\|proteobacteria\|deltaproteobacteria\|desulfovibrionales\|desulfovibrionaceae\|desulfovibrio\|desulfovibrio fairfieldensis | 48.45 | -2.73 | 0.000 | 0.000 |
| bacteria\|firmicutes\|negativicutes\|selenomonadales\|veillonellaceae\|dialister\|dialister succinatiphilus | 22.50 | 1.59 | 0.003 | 0.015 |
| bacteria\|firmicutes\|clostridia\|clostridiales\|clostridiales\|flavonifractor\|clostridium orbiscindens | 318.61 | 3.36 | 0.000 | 0.000 |
| bacteria\|bacteroidetes\|bacteroidia\|bacteroidales\|porphyromonadaceae\|butyricimonas\|butyricimonas sp. | 50.81 | -3.19 | 0.000 | 0.000 |
| bacteria\|bacteroidetes\|bacteroidia\|bacteroidales\|porphyromonadaceae\|odoribacter\|odoribacter splanchnicus | 165.32 | -4.55 | 0.000 | 0.000 |
| bacteria\|firmicutes\|clostridia\|clostridiales\|lachnospiraceae\|blautia\|blautia wexlerae | 210.91 | 0.92 | 0.007 | 0.030 |
| bacteria\|bacteroidetes\|bacteroidia\|bacteroidales\|porphyromonadaceae\|butyricimonas\|butyricimonas virosa | 105.01 | -3.70 | 0.000 | 0.000 |
| bacteria\|firmicutes\|clostridia\|clostridiales\|lachnospiraceae\|coprococcus\|coprococcus eutactus | 48.94 | -1.33 | 0.009 | 0.037 |
| bacteria\|bacteroidetes\|bacteroidia\|bacteroidales\|porphyromonadaceae\|paludibacter\|paludibacter spp. | 131.06 | -4.27 | 0.000 | 0.000 |
| bacteria\|firmicutes\|clostridia\|clostridiales\|ruminococcaceae\|oscillospira\|oscillospira spp..3 | 172.86 | -3.22 | 0.000 | 0.000 |
| bacteria\|bacteroidetes\|bacteroidia\|bacteroidales\|rikenellaceae\|alistipes\|alistipes indistinctus | 233.57 | -5.20 | 0.000 | 0.000 |
| bacteria\|firmicutes\|clostridia\|clostridiales\|clostridiaceae\|clostridium\|clostridium sp..6 | 100.10 | -1.97 | 0.003 | 0.015 |
| bacteria\|firmicutes\|clostridia\|clostridiales\|eubacteriaceae\|eubacterium\|eubacterium spp. | 24.60 | -3.84 | 0.000 | 0.000 |
| bacteria\|bacteroidetes\|bacteroidia\|bacteroidales\|prevotellaceae\|paraprevotella\|paraprevotella clara | 36.62 | -2.17 | 0.000 | 0.003 |
| bacteria\|bacteroidetes\|bacteroidia\|bacteroidales\|bacteroidaceae\|bacteroides\|bacteroides spp. | 176.43 | -3.25 | 0.000 | 0.000 |
| bacteria\|firmicutes\|clostridia\|clostridiales\|ruminococcaceae\|oscillospira\|oscillospira spp..4 | 86.04 | -3.71 | 0.000 | 0.000 |
| bacteria\|firmicutes\|clostridia\|clostridiales\|clostridiaceae\|clostridium\|clostridium sp..8 | 54.59 | 2.07 | 0.003 | 0.015 |
| bacteria\|firmicutes\|clostridia\|clostridiales\|clostridiaceae\|clostridium\|clostridium sp..10 | 41.30 | 1.77 | 0.003 | 0.015 |
| bacteria\|proteobacteria\|deltaproteobacteria\|desulfovibrionales\|desulfovibrionaceae\|desulfovibrio\|desulfovibrio piger | 17.61 | -2.44 | 0.000 | 0.003 |
| bacteria\|firmicutes\|erysipelotrichia\|erysipelotrichales\|erysipelotrichaceae\|faecalitalea\|eubacterium cylindroides | 71.72 | 4.07 | 0.000 | 0.000 |
| bacteria\|bacteroidetes\|bacteroidia\|bacteroidales\|porphyromonadaceae\|butyricimonas\|butyricimonas sp..1 | 90.15 | -3.89 | 0.000 | 0.000 |
| bacteria\|firmicutes\|clostridia\|clostridiales\|clostridiaceae\|clostridium\|clostridium sp..11 | 46.00 | -2.49 | 0.000 | 0.000 |
| bacteria\|bacteroidetes\|bacteroidia\|bacteroidales\|bacteroidaceae\|bacteroides\|bacteroides stercorirosoris | 46.39 | -1.76 | 0.005 | 0.022 |
| bacteria\|firmicutes\|clostridia\|clostridiales\|lachnospiraceae\|tyzzerella\|clostridium lactatifermentans | 38.74 | 2.51 | 0.000 | 0.002 |
| bacteria\|bacteroidetes\|bacteroidia\|bacteroidales\|rikenellaceae\|alistipes\|alistipes sp. | 118.96 | -3.71 | 0.000 | 0.000 |
| bacteria\|firmicutes\|clostridia\|clostridiales\|ruminococcaceae\|anaerotruncus\|anaerotruncus colihominis | 43.86 | 2.94 | 0.000 | 0.003 |
| bacteria\|firmicutes\|clostridia\|clostridiales\|ruminococcaceae\|ruminococcus\|ruminococcus callidus | 45.48 | -2.50 | 0.000 | 0.000 |
| bacteria\|firmicutes\|clostridia\|clostridiales\|eubacteriaceae\|eubacterium\|eubacterium spp..1 | 7.78 | -2.46 | 0.000 | 0.003 |
| bacteria\|bacteroidetes\|bacteroidia\|bacteroidales\|porphyromonadaceae\|paludibacter\|paludibacter spp..2 | 50.10 | -4.05 | 0.000 | 0.000 |
| bacteria\|firmicutes\|clostridia\|clostridiales\|ruminococcaceae\|ruminiclostridium\|clostridium leptum.1 | 51.85 | 3.04 | 0.000 | 0.000 |
| bacteria\|firmicutes\|clostridia\|clostridiales\|lachnospiraceae\|lachnoclostridium\|clostridium hathewayi | 110.17 | 2.15 | 0.000 | 0.003 |
| bacteria\|firmicutes\|clostridia\|clostridiales\|lachnospiraceae\|lachnoclostridium\|clostridium scindens | 83.88 | 2.84 | 0.000 | 0.000 |
| bacteria\|firmicutes\|clostridia\|clostridiales\|lachnospiraceae\|roseburia\|roseburia faecis | 16.52 | 1.89 | 0.002 | 0.013 |
| bacteria\|firmicutes\|clostridia\|clostridiales\|lachnospiraceae\|lachnobacterium\|lachnobacterium bovis | 32.80 | -2.00 | 0.000 | 0.003 |
| bacteria\|bacteroidetes\|bacteroidia\|bacteroidales\|porphyromonadaceae\|butyricimonas\|butyricimonas sp..2 | 37.94 | -3.15 | 0.000 | 0.001 |
| bacteria\|firmicutes\|clostridia\|clostridiales\|clostridiaceae\|clostridium\|clostridium spp..3 | 6.89 | -2.03 | 0.008 | 0.032 |
| bacteria\|proteobacteria\|alphaproteobacteria\|kopriimonadales\|kopriimonadaceae\|kopriimonas\|kopriimonas spp..1 | 6.32 | -2.75 | 0.005 | 0.021 |
| bacteria\|firmicutes\|clostridia\|clostridiales\|ruminococcaceae\|ruminiclostridium\|eubacterium siraeum | 35.75 | -2.11 | 0.008 | 0.033 |
| bacteria\|firmicutes\|clostridia\|clostridiales\|eubacteriaceae\|eubacterium\|eubacterium ruminantium | 13.24 | -1.83 | 0.005 | 0.021 |
| bacteria\|firmicutes\|clostridia\|clostridiales\|lachnospiraceae\|lachnoclostridium\|clostridium aldenense | 68.76 | 2.90 | 0.000 | 0.000 |
| bacteria\|firmicutes\|clostridia\|clostridiales\|clostridiaceae\|clostridium\|clostridium sp..14 | 23.57 | -2.56 | 0.001 | 0.006 |
| bacteria\|firmicutes\|clostridia\|clostridiales\|ruminococcaceae\|oscillospira\|oscillospira spp..5 | 29.72 | -4.18 | 0.000 | 0.000 |
| bacteria\|firmicutes\|negativicutes\|selenomonadales\|veillonellaceae\|allisonella\|allisonella spp. | 13.85 | 1.57 | 0.001 | 0.007 |
| bacteria\|firmicutes\|clostridia\|clostridiales\|lachnospiraceae\|tyzzerella\|clostridium lactatifermentans.1 | 34.56 | -2.91 | 0.000 | 0.000 |
| bacteria\|firmicutes\|clostridia\|clostridiales\|ruminococcaceae\|ruminococcus\|ruminococcus spp..3 | 24.35 | 1.84 | 0.014 | 0.049 |
| bacteria\|actinobacteria\|actinobacteria\|bifidobacteriales\|bifidobacteriaceae\|bifidobacterium\|bifidobacterium scardovii | 15.47 | 3.27 | 0.000 | 0.001 |
| bacteria\|firmicutes\|clostridia\|clostridiales\|ruminococcaceae\|oscillospira\|oscillospira spp..7 | 15.75 | 2.67 | 0.001 | 0.006 |
| bacteria\|firmicutes\|clostridia\|clostridiales\|lachnospiraceae\|dorea\|candidatus dorea massiliensis.1 | 7.71 | 1.47 | 0.006 | 0.026 |
| bacteria\|firmicutes\|clostridia\|clostridiales\|lachnospiraceae\|coprococcus\|coprococcus catus.1 | 21.36 | -1.99 | 0.003 | 0.014 |
| bacteria\|verrucomicrobia\|opitutae\|opitutales\|opitutaceae\|opitutus\|opitutus spp. | 5.15 | -4.47 | 0.004 | 0.020 |
| bacteria\|firmicutes\|clostridia\|clostridiales\|ruminococcaceae\|ruminococcus\|ruminococcus sp..3 | 35.65 | -2.61 | 0.000 | 0.000 |
| bacteria\|firmicutes\|clostridia\|clostridiales\|lachnospiraceae\|roseburia\|roseburia faecis.1 | 16.10 | 3.27 | 0.000 | 0.001 |
| bacteria\|firmicutes\|clostridia\|clostridiales\|clostridiaceae\|clostridium\|clostridium disporicum | 16.30 | 2.79 | 0.001 | 0.005 |
| bacteria\|firmicutes\|clostridia\|clostridiales\|ruminococcaceae\|oscillospira\|oscillospira spp..8 | 25.11 | -3.72 | 0.000 | 0.000 |
| bacteria\|bacteroidetes\|bacteroidia\|bacteroidales\|rikenellaceae\|alistipes\|alistipes indistinctus.1 | 10.87 | -4.32 | 0.000 | 0.001 |
| bacteria\|firmicutes\|clostridia\|clostridiales\|peptostreptococcaceae\|intestinibacter\|clostridium bartlettii | 18.95 | 3.66 | 0.000 | 0.000 |
| bacteria\|firmicutes\|clostridia\|clostridiales\|clostridiaceae\|clostridium\|clostridium sp..18 | 16.60 | -2.53 | 0.000 | 0.003 |
| bacteria\|firmicutes\|clostridia\|clostridiales\|ruminococcaceae\|ruminococcus\|ruminococcus sp..4 | 12.82 | -1.77 | 0.012 | 0.045 |
| bacteria\|firmicutes\|clostridia\|clostridiales\|clostridiaceae\|clostridium\|clostridium spp..6 | 11.97 | -2.09 | 0.000 | 0.001 |
| bacteria\|firmicutes\|clostridia\|clostridiales\|ruminococcaceae\|oscillospira\|oscillospira spp..9 | 7.18 | -3.38 | 0.000 | 0.003 |
| bacteria\|firmicutes\|clostridia\|clostridiales\|eubacteriaceae\|eubacterium\|eubacterium spp..3 | 10.76 | -4.40 | 0.000 | 0.001 |
| bacteria\|proteobacteria\|betaproteobacteria\|burkholderiales\|oxalobacteraceae\|oxalobacter\|oxalobacter spp. | 11.01 | -3.42 | 0.000 | 0.000 |
| bacteria\|synergistetes\|synergistia\|synergistales\|synergistaceae\|pyramidobacter\|pyramidobacter piscolens | 4.85 | 3.53 | 0.008 | 0.033 |
| bacteria\|firmicutes\|clostridia\|clostridiales\|clostridiaceae\|clostridium\|clostridium sp..21 | 7.73 | 2.67 | 0.000 | 0.003 |
| bacteria\|firmicutes\|clostridia\|clostridiales\|ruminococcaceae\|ruminococcus\|ruminococcus spp..4 | 5.15 | -2.75 | 0.011 | 0.042 |
| bacteria\|bacteroidetes\|bacteroidia\|bacteroidales\|porphyromonadaceae\|butyricimonas\|butyricimonas sp..5 | 5.62 | -1.53 | 0.013 | 0.049 |
| bacteria\|firmicutes\|clostridia\|clostridiales\|lachnospiraceae\|lachnoclostridium\|clostridium asparagiforme | 11.63 | -2.86 | 0.000 | 0.000 |
| bacteria\|firmicutes\|clostridia\|clostridiales\|lachnospiraceae\|lachnoclostridium\|clostridium xylanolyticum.1 | 267.71 | -1.61 | 0.003 | 0.014 |
| bacteria\|firmicutes\|clostridia\|clostridiales\|ruminococcaceae\|ruminococcus\|ruminococcus sp..6 | 27.78 | 2.96 | 0.000 | 0.000 |
| bacteria\|firmicutes\|clostridia\|clostridiales\|ruminococcaceae\|acetanaerobacterium\|acetanaerobacterium elongatum.1 | 4.93 | -3.42 | 0.001 | 0.007 |
| bacteria\|firmicutes\|clostridia\|clostridiales\|ruminococcaceae\|ruminiclostridium\|eubacterium siraeum.1 | 3.65 | -2.52 | 0.005 | 0.024 |
| bacteria\|firmicutes\|clostridia\|clostridiales\|clostridiaceae\|clostridium\|clostridium sp..23 | 7.08 | -3.11 | 0.002 | 0.013 |
| bacteria\|bacteroidetes\|bacteroidia\|bacteroidales\|porphyromonadaceae\|paludibacter\|paludibacter spp..5 | 4.85 | -2.73 | 0.002 | 0.011 |
| bacteria\|firmicutes\|clostridia\|clostridiales\|lachnospiraceae\|lachnoclostridium\|clostridium lavalense | 94.98 | 2.73 | 0.000 | 0.000 |
| bacteria\|bacteroidetes\|bacteroidia\|bacteroidales\|rikenellaceae\|alistipes\|candidatus alistipes marseilloanorexicus | 5.15 | -4.25 | 0.000 | 0.000 |
| bacteria\|firmicutes\|bacilli\|bacillales\|bacillaceae\|bacillus\|bacillus spp..1 | 4.27 | -2.99 | 0.006 | 0.026 |
| bacteria\|bacteroidetes\|bacteroidia\|bacteroidales\|rikenellaceae\|alistipes\|alistipes finegoldii.3 | 20.98 | 1.82 | 0.000 | 0.002 |
| bacteria\|firmicutes\|clostridia\|clostridiales\|clostridiaceae\|clostridium\|clostridium sp..24 | 6.32 | -3.26 | 0.000 | 0.002 |
| bacteria\|firmicutes\|negativicutes\|selenomonadales\|veillonellaceae\|veillonella\|veillonella sp. | 9.74 | 4.17 | 0.000 | 0.001 |
| bacteria\|firmicutes\|clostridia\|clostridiales\|ruminococcaceae\|oscillospira\|oscillospira spp..11 | 1.61 | -2.32 | 0.010 | 0.040 |
| bacteria\|firmicutes\|clostridia\|clostridiales\|ruminococcaceae\|oscillospira\|oscillospira spp..12 | 65.42 | -3.77 | 0.000 | 0.000 |
| bacteria\|firmicutes\|clostridia\|clostridiales\|ruminococcaceae\|anaerotruncus\|anaerotruncus colihominis.2 | 3.04 | -2.76 | 0.000 | 0.002 |
| bacteria\|firmicutes\|clostridia\|clostridiales\|lachnospiraceae\|tyzzerella\|clostridium neopropionicum.1 | 6.91 | 2.30 | 0.003 | 0.014 |
| bacteria\|firmicutes\|clostridia\|clostridiales\|lachnospiraceae\|marvinbryantia\|bryantella formatexigens | 6.56 | -2.98 | 0.000 | 0.003 |
| bacteria\|firmicutes\|erysipelotrichia\|erysipelotrichales\|erysipelotrichaceae\|turicibacter\|turicibacter spp..4 | 6.77 | 2.64 | 0.007 | 0.028 |
| bacteria\|bacteroidetes\|bacteroidia\|bacteroidales\|bacteroidaceae\|bacteroides\|bacteroides uniformis.2 | 978.10 | 1.88 | 0.002 | 0.011 |
| bacteria\|bacteroidetes\|bacteroidia\|bacteroidales\|bacteroidaceae\|bacteroides\|bacteroides thetaiotaomicron.1 | 56.47 | 1.90 | 0.001 | 0.009 |
| bacteria\|firmicutes\|bacilli\|bacillales\|bacillaceae\|bacillus\|bacillus spp..2 | 4.24 | -3.27 | 0.006 | 0.027 |
| bacteria\|firmicutes\|clostridia\|clostridiales\|clostridiaceae\|clostridium\|clostridium sp..26 | 2.76 | -2.26 | 0.006 | 0.027 |
| Other\|Other\|Other\|Other\|Other\|Other\|Other | 2.25 | 2.02 | 0.011 | 0.041 |
| bacteria\|firmicutes\|clostridia\|clostridiales\|lachnospiraceae\|coprococcus\|coprococcus catus.2 | 3.41 | -2.85 | 0.002 | 0.011 |
| bacteria\|firmicutes\|clostridia\|clostridiales\|lachnospiraceae\|lachnoclostridium\|clostridium symbiosum | 118.37 | 2.91 | 0.000 | 0.000 |
| bacteria\|firmicutes\|clostridia\|clostridiales\|ruminococcaceae\|anaerofilum\|anaerofilum spp. | 4.08 | -4.12 | 0.001 | 0.006 |
| bacteria\|firmicutes\|clostridia\|clostridiales\|ruminococcaceae\|ruminococcus\|ruminococcus flavefaciens.2 | 4.36 | -2.86 | 0.003 | 0.014 |
| bacteria\|bacteroidetes\|bacteroidia\|bacteroidales\|rikenellaceae\|alistipes\|alistipes shahii | 10.72 | -1.87 | 0.002 | 0.012 |
| bacteria\|firmicutes\|clostridia\|clostridiales\|ruminococcaceae\|oscillospira\|oscillospira spp..14 | 5.94 | -3.36 | 0.001 | 0.006 |
| bacteria\|firmicutes\|clostridia\|clostridiales\|eubacteriaceae\|eubacterium\|eubacterium sp..1 | 3.30 | -3.12 | 0.000 | 0.003 |
| bacteria\|bacteroidetes\|bacteroidia\|bacteroidales\|porphyromonadaceae\|barnesiella\|barnesiella intestinihominis.1 | 6.73 | -2.68 | 0.000 | 0.003 |
| bacteria\|firmicutes\|clostridia\|clostridiales\|ruminococcaceae\|ethanoligenens\|ethanoligenens spp. | 3.81 | -2.73 | 0.006 | 0.026 |
| bacteria\|firmicutes\|clostridia\|clostridiales\|lachnospiraceae\|blautia\|blautia producta.1 | 4.14 | -3.10 | 0.000 | 0.001 |
| bacteria\|firmicutes\|clostridia\|clostridiales\|clostridiaceae\|clostridium\|clostridium spp..14 | 4.60 | -2.39 | 0.004 | 0.018 |
| bacteria\|firmicutes\|clostridia\|clostridiales\|clostridiales\|flavonifractor\|clostridium orbiscindens.3 | 4.17 | -3.29 | 0.002 | 0.013 |
| bacteria\|bacteroidetes\|bacteroidia\|bacteroidales\|rikenellaceae\|alistipes\|alistipes putredinis.1 | 3.63 | -2.92 | 0.011 | 0.043 |
| bacteria\|bacteroidetes\|bacteroidia\|bacteroidales\|rikenellaceae\|alistipes\|alistipes finegoldii.4 | 10.59 | -4.65 | 0.000 | 0.000 |
| bacteria\|firmicutes\|clostridia\|clostridiales\|ruminococcaceae\|oscillospira\|oscillospira spp..15 | 9.11 | -3.59 | 0.000 | 0.000 |
| bacteria\|actinobacteria\|actinobacteria\|bifidobacteriales\|bifidobacteriaceae\|bifidobacterium\|bifidobacterium catenulatum | 3.87 | 3.81 | 0.004 | 0.018 |
| bacteria\|firmicutes\|clostridia\|clostridiales\|ruminococcaceae\|ruminiclostridium\|clostridium straminisolvens | 1.68 | -3.06 | 0.004 | 0.019 |
| bacteria\|proteobacteria\|alphaproteobacteria\|rhodospirillales\|acetobacteraceae\|saccharibacter\|saccharibacter spp. | 3.49 | 4.10 | 0.000 | 0.002 |
| bacteria\|actinobacteria\|actinobacteria\|bifidobacteriales\|bifidobacteriaceae\|bifidobacterium\|bifidobacterium catenulatum.1 | 27.99 | 4.17 | 0.000 | 0.000 |
| bacteria\|firmicutes\|clostridia\|clostridiales\|clostridiaceae\|clostridium\|clostridium spp..15 | 2.40 | -3.27 | 0.002 | 0.014 |
| bacteria\|firmicutes\|clostridia\|clostridiales\|ruminococcaceae\|oscillospira\|oscillospira spp..18 | 11.31 | 3.69 | 0.000 | 0.000 |
| bacteria\|firmicutes\|clostridia\|clostridiales\|clostridiaceae\|clostridium\|clostridium sp..29 | 3.04 | -3.90 | 0.000 | 0.003 |
| bacteria\|firmicutes\|clostridia\|clostridiales\|ruminococcaceae\|oscillospira\|oscillospira spp..19 | 6.07 | 2.17 | 0.001 | 0.008 |
| bacteria\|firmicutes\|clostridia\|clostridiales\|oscillospiraceae\|oscillibacter\|oscillibacter sp. | 3.62 | -4.19 | 0.000 | 0.000 |
| bacteria\|firmicutes\|clostridia\|clostridiales\|clostridiaceae\|clostridium\|clostridium sp..32 | 2.32 | -3.99 | 0.010 | 0.040 |
| bacteria\|firmicutes\|clostridia\|clostridiales\|lachnospiraceae\|blautia\|ruminococcus gnavus.3 | 21.43 | 2.32 | 0.000 | 0.000 |
| bacteria\|firmicutes\|clostridia\|clostridiales\|clostridiales\|flavonifractor\|clostridium orbiscindens.4 | 3.61 | 4.05 | 0.000 | 0.000 |
| bacteria\|bacteroidetes\|bacteroidia\|bacteroidales\|porphyromonadaceae\|butyricimonas\|butyricimonas sp..7 | 8.16 | -2.68 | 0.000 | 0.003 |
| bacteria\|firmicutes\|clostridia\|clostridiales\|eubacteriaceae\|alkalibacter\|alkalibacter spp..1 | 3.41 | -2.91 | 0.001 | 0.009 |
| bacteria\|bacteroidetes\|bacteroidia\|bacteroidales\|porphyromonadaceae\|paludibacter\|paludibacter spp..6 | 2.09 | -2.85 | 0.001 | 0.006 |
| bacteria\|actinobacteria\|actinobacteria\|bifidobacteriales\|bifidobacteriaceae\|bifidobacterium\|bifidobacterium dentium | 267.05 | 5.11 | 0.000 | 0.000 |
| bacteria\|firmicutes\|clostridia\|clostridiales\|ruminococcaceae\|oscillospira\|oscillospira spp..22 | 2.11 | -3.14 | 0.011 | 0.040 |
| bacteria\|firmicutes\|clostridia\|clostridiales\|lachnospiraceae\|fusicatenibacter\|fusicatenibacter saccharivorans.2 | 3.03 | -3.03 | 0.001 | 0.006 |
| bacteria\|firmicutes\|clostridia\|clostridiales\|eubacteriaceae\|eubacterium\|eubacterium rectale.5 | 6.77 | -2.10 | 0.001 | 0.004 |
| bacteria\|bacteroidetes\|bacteroidia\|bacteroidales\|porphyromonadaceae\|barnesiella\|barnesiella intestinihominis.3 | 3.27 | -4.24 | 0.004 | 0.021 |
| bacteria\|firmicutes\|clostridia\|clostridiales\|lachnospiraceae\|blautia\|blautia producta.3 | 5.44 | 1.64 | 0.002 | 0.012 |
| bacteria\|bacteroidetes\|bacteroidia\|bacteroidales\|porphyromonadaceae\|paludibacter\|paludibacter spp..7 | 7.52 | -3.79 | 0.000 | 0.000 |
| bacteria\|firmicutes\|clostridia\|clostridiales\|ruminococcaceae\|oscillospira\|oscillospira spp..23 | 2.03 | -3.28 | 0.000 | 0.003 |
| bacteria\|firmicutes\|clostridia\|clostridiales\|clostridiaceae\|clostridium\|clostridium sp..35 | 1.83 | -2.63 | 0.007 | 0.030 |
| bacteria\|firmicutes\|clostridia\|clostridiales\|eubacteriaceae\|eubacterium\|eubacterium rectale.6 | 2.59 | -2.94 | 0.003 | 0.016 |
| bacteria\|firmicutes\|clostridia\|clostridiales\|lachnospiraceae\|lachnoclostridium\|clostridium polysaccharolyticum.1 | 2.11 | -2.32 | 0.004 | 0.019 |
| bacteria\|bacteroidetes\|bacteroidia\|bacteroidales\|porphyromonadaceae\|barnesiella\|barnesiella intestinihominis.4 | 11.17 | -5.61 | 0.000 | 0.000 |
| bacteria\|firmicutes\|clostridia\|clostridiales\|clostridiaceae\|clostridium\|clostridium sp..36 | 2.73 | -3.77 | 0.008 | 0.034 |
| bacteria\|firmicutes\|clostridia\|clostridiales\|eubacteriaceae\|eubacterium\|eubacterium rectale.7 | 2.37 | -2.77 | 0.005 | 0.024 |
| bacteria\|firmicutes\|clostridia\|clostridiales\|ruminococcaceae\|oscillospira\|oscillospira spp..26 | 3.02 | -3.62 | 0.007 | 0.028 |
| bacteria\|firmicutes\|clostridia\|clostridiales\|eubacteriaceae\|eubacterium\|eubacterium rectale.8 | 9.22 | -1.85 | 0.012 | 0.045 |
| bacteria\|firmicutes\|clostridia\|clostridiales\|ruminococcaceae\|ruminococcus\|ruminococcus spp..11 | 1.56 | -2.89 | 0.007 | 0.030 |
| bacteria\|firmicutes\|clostridia\|clostridiales\|ruminococcaceae\|oscillospira\|oscillospira spp..31 | 84.58 | -3.56 | 0.000 | 0.000 |
| bacteria\|firmicutes\|clostridia\|clostridiales\|ruminococcaceae\|oscillospira\|oscillospira spp..32 | 3.22 | -2.03 | 0.008 | 0.034 |
| bacteria\|bacteroidetes\|bacteroidia\|bacteroidales\|bacteroidaceae\|bacteroides\|bacteroides uniformis.7 | 387.68 | 1.86 | 0.000 | 0.003 |
| bacteria\|firmicutes\|clostridia\|clostridiales\|lachnospiraceae\|blautia\|ruminococcus gnavus.5 | 9.20 | 2.80 | 0.000 | 0.001 |
| bacteria\|firmicutes\|clostridia\|clostridiales\|ruminococcaceae\|anaerofilum\|anaerofilum spp..2 | 1.77 | -2.74 | 0.010 | 0.040 |
|  |  |  |  |  |
| bacteria\|bacteroidetes\|bacteroidia\|bacteroidales\|rikenellaceae\|alistipes\|alistipes sp..2 | 46.31 | -3.84 | 0.000 | 0.000 |
| bacteria\|firmicutes\|clostridia\|clostridiales\|clostridiaceae\|clostridium\|clostridium sp..47 | 10.34 | -4.47 | 0.001 | 0.008 |
| bacteria\|firmicutes\|clostridia\|clostridiales\|ruminococcaceae\|oscillospira\|oscillospira spp..34 | 73.40 | 3.01 | 0.000 | 0.000 |
| bacteria\|firmicutes\|clostridia\|clostridiales\|ruminococcaceae\|oscillospira\|oscillospira spp..35 | 2.36 | -3.73 | 0.001 | 0.009 |
| bacteria\|bacteroidetes\|bacteroidia\|bacteroidales\|bacteroidaceae\|bacteroides\|bacteroides dorei | 94.21 | 2.09 | 0.000 | 0.000 |
| bacteria\|firmicutes\|clostridia\|clostridiales\|ruminococcaceae\|oscillospira\|oscillospira spp..36 | 9.94 | -3.87 | 0.000 | 0.001 |
| bacteria\|firmicutes\|clostridia\|clostridiales\|lachnospiraceae\|lachnoclostridium\|clostridium lavalense.1 | 20.22 | 2.41 | 0.000 | 0.001 |
| bacteria\|candidatus saccharibacteria\|candidatus saccharibacteria\|candidatus saccharibacteria\|candidatus saccharibacteria\|candidatus saccharimonas\|candidatus saccharimonas aalborgensis | 2.67 | 3.71 | 0.003 | 0.015 |
| bacteria\|firmicutes\|clostridia\|clostridiales\|lachnospiraceae\|lachnoclostridium\|clostridium bolteae.1 | 12.82 | -1.78 | 0.008 | 0.034 |
| bacteria\|firmicutes\|erysipelotrichia\|erysipelotrichales\|erysipelotrichaceae\|holdemanella\|eubacterium biforme.2 | 7.30 | -3.66 | 0.000 | 0.001 |
| bacteria\|firmicutes\|clostridia\|clostridiales\|lachnospiraceae\|blautia\|ruminococcus gnavus.6 | 7.91 | 1.52 | 0.011 | 0.041 |
| bacteria\|firmicutes\|clostridia\|clostridiales\|ruminococcaceae\|subdoligranulum\|subdoligranulum spp..12 | 5.73 | 3.44 | 0.000 | 0.002 |
| bacteria\|firmicutes\|clostridia\|clostridiales\|ruminococcaceae\|oscillospira\|oscillospira spp..38 | 2.48 | -4.10 | 0.000 | 0.000 |
| bacteria\|firmicutes\|clostridia\|clostridiales\|clostridiaceae\|clostridium\|clostridium spp..21 | 1.33 | -2.85 | 0.013 | 0.046 |
| bacteria\|bacteroidetes\|bacteroidia\|bacteroidales\|bacteroidaceae\|bacteroides\|bacteroides spp..2 | 391.38 | 1.76 | 0.000 | 0.003 |
| bacteria\|firmicutes\|clostridia\|clostridiales\|lachnospiraceae\|blautia\|ruminococcus gnavus.7 | 9.65 | 3.09 | 0.002 | 0.009 |
| bacteria\|firmicutes\|clostridia\|clostridiales\|clostridiaceae\|clostridium\|clostridium sp..57 | 1.23 | -3.07 | 0.014 | 0.049 |
| bacteria\|bacteroidetes\|bacteroidia\|bacteroidales\|bacteroidaceae\|bacteroides\|bacteroides fragilis.1 | 6.24 | 2.70 | 0.003 | 0.014 |
| bacteria\|firmicutes\|negativicutes\|selenomonadales\|veillonellaceae\|dialister\|dialister invisus.1 | 5.22 | 2.53 | 0.008 | 0.034 |
| bacteria\|bacteroidetes\|bacteroidia\|bacteroidales\|porphyromonadaceae\|barnesiella\|barnesiella intestinihominis.7 | 9.08 | -5.74 | 0.000 | 0.002 |
| bacteria\|bacteroidetes\|bacteroidia\|bacteroidales\|porphyromonadaceae\|barnesiella\|barnesiella intestinihominis.8 | 8.82 | -4.79 | 0.000 | 0.001 |
| bacteria\|firmicutes\|clostridia\|clostridiales\|ruminococcaceae\|oscillospira\|oscillospira spp..44 | 2.76 | -3.28 | 0.001 | 0.005 |
| bacteria\|bacteroidetes\|bacteroidia\|bacteroidales\|bacteroidaceae\|bacteroides\|bacteroides uniformis.10 | 180.38 | 2.12 | 0.000 | 0.003 |
| bacteria\|bacteroidetes\|bacteroidia\|bacteroidales\|bacteroidaceae\|bacteroides\|bacteroides vulgatus.3 | 11.84 | 2.21 | 0.001 | 0.009 |
| bacteria\|firmicutes\|clostridia\|clostridiales\|lachnospiraceae\|roseburia\|roseburia faecis.8 | 2.95 | 2.47 | 0.001 | 0.007 |
| bacteria\|bacteroidetes\|bacteroidia\|bacteroidales\|bacteroidaceae\|bacteroides\|bacteroides vulgatus.4 | 6.47 | 3.63 | 0.000 | 0.001 |
| bacteria\|firmicutes\|clostridia\|clostridiales\|ruminococcaceae\|oscillospira\|oscillospira spp..50 | 1.22 | -2.80 | 0.001 | 0.006 |
| bacteria\|firmicutes\|clostridia\|clostridiales\|ruminococcaceae\|oscillospira\|oscillospira spp..51 | 2.73 | -2.86 | 0.005 | 0.024 |
| bacteria\|bacteroidetes\|bacteroidia\|bacteroidales\|bacteroidaceae\|bacteroides\|bacteroides dorei.1 | 92.80 | 2.68 | 0.000 | 0.001 |
| bacteria\|firmicutes\|clostridia\|clostridiales\|ruminococcaceae\|oscillospira\|oscillospira spp..52 | 9.17 | -2.59 | 0.007 | 0.030 |
| bacteria\|firmicutes\|clostridia\|clostridiales\|lachnospiraceae\|dorea\|candidatus dorea massiliensis.2 | 6.93 | -2.87 | 0.000 | 0.001 |
| bacteria\|firmicutes\|clostridia\|clostridiales\|ruminococcaceae\|oscillospira\|oscillospira spp..54 | 6.71 | 3.68 | 0.000 | 0.000 |
| bacteria\|bacteroidetes\|bacteroidia\|bacteroidales\|rikenellaceae\|alistipes\|alistipes shahii.1 | 57.22 | -2.58 | 0.000 | 0.001 |
| bacteria\|firmicutes\|clostridia\|clostridiales\|eubacteriaceae\|eubacterium\|eubacterium rectale.16 | 14.17 | 2.82 | 0.000 | 0.001 |
| bacteria\|firmicutes\|clostridia\|clostridiales\|ruminococcaceae\|oscillospira\|oscillospira spp..55 | 3.71 | -1.86 | 0.006 | 0.026 |
| bacteria\|bacteroidetes\|bacteroidia\|bacteroidales\|bacteroidaceae\|bacteroides\|bacteroides dorei.2 | 5213.06 | 2.56 | 0.000 | 0.002 |
| bacteria\|firmicutes\|clostridia\|clostridiales\|clostridiaceae\|clostridium\|clostridium sp..69 | 1.69 | -3.50 | 0.005 | 0.022 |
| bacteria\|firmicutes\|clostridia\|clostridiales\|ruminococcaceae\|ruminococcus\|ruminococcus spp..20 | 7.81 | -1.90 | 0.003 | 0.016 |
| bacteria\|bacteroidetes\|bacteroidia\|bacteroidales\|bacteroidaceae\|bacteroides\|bacteroides dorei.3 | 11.09 | 2.86 | 0.001 | 0.005 |
| bacteria\|firmicutes\|clostridia\|clostridiales\|eubacteriaceae\|eubacterium\|eubacterium rectale.18 | 1.68 | 3.15 | 0.003 | 0.017 |
| bacteria\|firmicutes\|clostridia\|clostridiales\|ruminococcaceae\|faecalibacterium\|faecalibacterium prausnitzii.12 | 3.22 | -3.29 | 0.007 | 0.031 |
| bacteria\|bacteroidetes\|bacteroidia\|bacteroidales\|bacteroidaceae\|bacteroides\|bacteroides finegoldii.1 | 6.02 | 2.01 | 0.001 | 0.005 |
| bacteria\|bacteroidetes\|bacteroidia\|bacteroidales\|porphyromonadaceae\|paludibacter\|paludibacter spp..13 | 2.04 | -3.00 | 0.005 | 0.024 |
| bacteria\|bacteroidetes\|bacteroidia\|bacteroidales\|bacteroidaceae\|bacteroides\|bacteroides uniformis.15 | 3.14 | -3.51 | 0.001 | 0.005 |
| bacteria\|proteobacteria\|betaproteobacteria\|burkholderiales\|sutterellaceae\|sutterella\|sutterella parvirubra.1 | 1.26 | 3.23 | 0.010 | 0.039 |
| bacteria\|firmicutes\|clostridia\|clostridiales\|ruminococcaceae\|oscillospira\|oscillospira spp..64 | 7.99 | -2.24 | 0.001 | 0.005 |
| bacteria\|firmicutes\|clostridia\|clostridiales\|clostridiaceae\|clostridium\|clostridium sp..75 | 23.22 | -2.89 | 0.000 | 0.000 |
| bacteria\|firmicutes\|clostridia\|clostridiales\|ruminococcaceae\|oscillospira\|oscillospira spp..65 | 41.78 | 2.74 | 0.000 | 0.003 |
| bacteria\|bacteroidetes\|bacteroidia\|bacteroidales\|bacteroidaceae\|bacteroides\|bacteroides vulgatus.5 | 11.94 | 1.20 | 0.006 | 0.028 |
| bacteria\|bacteroidetes\|bacteroidia\|bacteroidales\|rikenellaceae\|alistipes\|alistipes finegoldii.5 | 279.36 | 3.21 | 0.000 | 0.003 |
| bacteria\|bacteroidetes\|bacteroidia\|bacteroidales\|bacteroidaceae\|bacteroides\|bacteroides thetaiotaomicron.5 | 26.70 | 3.07 | 0.000 | 0.000 |
| bacteria\|actinobacteria\|actinobacteria\|coriobacteriales\|coriobacteriaceae\|atopobium\|atopobium parvulum | 0.86 | 2.89 | 0.011 | 0.042 |
| bacteria\|firmicutes\|clostridia\|clostridiales\|ruminococcaceae\|oscillospira\|oscillospira spp..70 | 17.54 | 3.03 | 0.000 | 0.001 |
| bacteria\|firmicutes\|clostridia\|clostridiales\|ruminococcaceae\|oscillospira\|oscillospira spp..72 | 2.81 | -3.56 | 0.009 | 0.036 |
| bacteria\|firmicutes\|clostridia\|clostridiales\|lachnospiraceae\|roseburia\|roseburia faecis.11 | 3.34 | -2.97 | 0.003 | 0.017 |
| bacteria\|firmicutes\|clostridia\|clostridiales\|ruminococcaceae\|oscillospira\|oscillospira spp..74 | 1.47 | -2.71 | 0.010 | 0.038 |
| bacteria\|bacteroidetes\|bacteroidia\|bacteroidales\|bacteroidaceae\|bacteroides\|bacteroides ovatus.2 | 63.20 | 1.49 | 0.014 | 0.049 |
| bacteria\|firmicutes\|clostridia\|clostridiales\|ruminococcaceae\|ruminococcus\|ruminococcus sp..22 | 13.07 | 1.97 | 0.003 | 0.016 |
| bacteria\|firmicutes\|clostridia\|clostridiales\|lachnospiraceae\|blautia\|ruminococcus torques.3 | 2.87 | -2.25 | 0.009 | 0.035 |
| bacteria\|bacteroidetes\|bacteroidia\|bacteroidales\|bacteroidaceae\|bacteroides\|bacteroides massiliensis.3 | 56.02 | -2.28 | 0.000 | 0.003 |
| bacteria\|bacteroidetes\|bacteroidia\|bacteroidales\|rikenellaceae\|alistipes\|alistipes putredinis.4 | 5.18 | -2.52 | 0.002 | 0.009 |
| bacteria\|firmicutes\|clostridia\|clostridiales\|ruminococcaceae\|oscillospira\|oscillospira spp..77 | 1.88 | 2.79 | 0.011 | 0.040 |
| bacteria\|firmicutes\|negativicutes\|selenomonadales\|acidaminococcaceae\|acidaminococcus\|acidaminococcus intestini.3 | 3.26 | 1.77 | 0.005 | 0.023 |
| bacteria\|bacteroidetes\|bacteroidia\|bacteroidales\|bacteroidaceae\|bacteroides\|bacteroides thetaiotaomicron.7 | 221.08 | 1.77 | 0.000 | 0.003 |
| bacteria\|firmicutes\|clostridia\|clostridiales\|clostridiaceae\|clostridium\|clostridium sp..98 | 2.32 | -3.50 | 0.001 | 0.005 |
| bacteria\|firmicutes\|clostridia\|clostridiales\|clostridiaceae\|clostridium\|clostridium sp..99 | 2.80 | -3.31 | 0.003 | 0.014 |
| bacteria\|actinobacteria\|actinobacteria\|bifidobacteriales\|bifidobacteriaceae\|bifidobacterium\|bifidobacterium catenulatum.3 | 253.66 | 4.47 | 0.000 | 0.000 |
| bacteria\|bacteroidetes\|bacteroidia\|bacteroidales\|bacteroidaceae\|bacteroides\|bacteroides thetaiotaomicron.8 | 271.61 | 2.04 | 0.000 | 0.001 |
| bacteria\|firmicutes\|clostridia\|clostridiales\|eubacteriaceae\|eubacterium\|eubacterium spp..5 | 2.49 | -2.66 | 0.002 | 0.013 |
| bacteria\|bacteroidetes\|bacteroidia\|bacteroidales\|bacteroidaceae\|bacteroides\|bacteroides ovatus.3 | 9.83 | 2.61 | 0.003 | 0.016 |
| bacteria\|firmicutes\|clostridia\|clostridiales\|ruminococcaceae\|oscillospira\|oscillospira spp..94 | 1.25 | 2.62 | 0.009 | 0.036 |
| bacteria\|bacteroidetes\|bacteroidia\|bacteroidales\|bacteroidaceae\|bacteroides\|bacteroides stercoris.5 | 147.37 | 3.42 | 0.000 | 0.000 |
| bacteria\|bacteroidetes\|bacteroidia\|bacteroidales\|bacteroidaceae\|bacteroides\|bacteroides ovatus.5 | 9.04 | 2.30 | 0.003 | 0.015 |
| bacteria\|bacteroidetes\|bacteroidia\|bacteroidales\|bacteroidaceae\|bacteroides\|bacteroides sp..2 | 366.23 | 1.83 | 0.000 | 0.000 |
| bacteria\|firmicutes\|clostridia\|clostridiales\|eubacteriaceae\|eubacterium\|eubacterium spp..7 | 8.64 | -4.82 | 0.000 | 0.000 |
| bacteria\|firmicutes\|clostridia\|clostridiales\|ruminococcaceae\|oscillospira\|oscillospira spp..103 | 18.91 | -5.46 | 0.000 | 0.000 |
| bacteria\|firmicutes\|clostridia\|clostridiales\|ruminococcaceae\|oscillospira\|oscillospira spp..104 | 1.86 | -3.15 | 0.001 | 0.005 |
| bacteria\|firmicutes\|clostridia\|clostridiales\|ruminococcaceae\|oscillospira\|oscillospira spp..105 | 7.09 | -4.28 | 0.000 | 0.000 |
| bacteria\|firmicutes\|clostridia\|clostridiales\|ruminococcaceae\|oscillospira\|oscillospira spp..108 | 2.50 | -3.13 | 0.000 | 0.003 |
| bacteria\|bacteroidetes\|bacteroidia\|bacteroidales\|bacteroidaceae\|bacteroides\|bacteroides salyersiae.1 | 306.38 | 2.61 | 0.000 | 0.001 |
| bacteria\|firmicutes\|clostridia\|clostridiales\|lachnospiraceae\|eisenbergiella\|eisenbergiella tayi.1 | 8.76 | 2.73 | 0.001 | 0.009 |
| bacteria\|bacteroidetes\|bacteroidia\|bacteroidales\|rikenellaceae\|alistipes\|alistipes putredinis.5 | 1.51 | -3.31 | 0.002 | 0.009 |
| bacteria\|firmicutes\|erysipelotrichia\|erysipelotrichales\|erysipelotrichaceae\|faecalitalea\|eubacterium cylindroides.3 | 2.91 | 4.07 | 0.002 | 0.012 |
| bacteria\|firmicutes\|clostridia\|clostridiales\|ruminococcaceae\|oscillospira\|oscillospira spp..111 | 5.46 | -2.51 | 0.000 | 0.004 |
| bacteria\|bacteroidetes\|bacteroidia\|bacteroidales\|rikenellaceae\|alistipes\|alistipes indistinctus.7 | 3.03 | -4.36 | 0.003 | 0.014 |
| bacteria\|bacteroidetes\|bacteroidia\|bacteroidales\|bacteroidaceae\|bacteroides\|bacteroides uniformis.24 | 26.84 | 1.35 | 0.010 | 0.039 |
| bacteria\|firmicutes\|clostridia\|clostridiales\|ruminococcaceae\|oscillospira\|oscillospira spp..118 | 3.59 | 2.86 | 0.000 | 0.001 |
| bacteria\|bacteroidetes\|bacteroidia\|bacteroidales\|bacteroidaceae\|bacteroides\|bacteroides finegoldii.2 | 8.82 | 1.38 | 0.009 | 0.035 |
| bacteria\|bacteroidetes\|bacteroidia\|bacteroidales\|bacteroidaceae\|bacteroides\|bacteroides sp..3 | 69.71 | 1.80 | 0.004 | 0.019 |
| bacteria\|bacteroidetes\|bacteroidia\|bacteroidales\|porphyromonadaceae\|paludibacter\|paludibacter spp..19 | 6.19 | -2.19 | 0.010 | 0.038 |
| bacteria\|bacteroidetes\|bacteroidia\|bacteroidales\|bacteroidaceae\|bacteroides\|bacteroides sp..4 | 1221.93 | 2.07 | 0.000 | 0.003 |
| bacteria\|firmicutes\|negativicutes\|selenomonadales\|veillonellaceae\|veillonella\|veillonella sp..1 | 16.54 | 3.22 | 0.000 | 0.001 |
| bacteria\|bacteroidetes\|bacteroidia\|bacteroidales\|porphyromonadaceae\|butyricimonas\|butyricimonas virosa.5 | 4.50 | -3.44 | 0.006 | 0.026 |
| bacteria\|proteobacteria\|gammaproteobacteria\|enterobacteriales\|enterobacteriaceae\|shigella\|shigella spp. | 32.81 | 2.62 | 0.000 | 0.003 |
| bacteria\|firmicutes\|clostridia\|clostridiales\|eubacteriaceae\|eubacterium\|eubacterium rectale.32 | 19.11 | 2.41 | 0.000 | 0.002 |
| bacteria\|bacteroidetes\|bacteroidia\|bacteroidales\|porphyromonadaceae\|paludibacter\|paludibacter spp..21 | 13.04 | -3.13 | 0.000 | 0.000 |
| bacteria\|firmicutes\|clostridia\|clostridiales\|lachnospiraceae\|lachnoclostridium\|clostridium xylanolyticum.4 | 5.19 | -2.50 | 0.004 | 0.020 |
| bacteria\|firmicutes\|clostridia\|clostridiales\|clostridiaceae\|clostridium\|clostridium sp..122 | 2.08 | -2.78 | 0.004 | 0.021 |
| bacteria\|firmicutes\|clostridia\|clostridiales\|lachnospiraceae\|eisenbergiella\|eisenbergiella tayi.2 | 19.61 | 2.12 | 0.009 | 0.037 |
| bacteria\|bacteroidetes\|bacteroidia\|bacteroidales\|bacteroidaceae\|bacteroides\|bacteroides dorei.4 | 45.31 | 2.33 | 0.000 | 0.002 |
| bacteria\|firmicutes\|clostridia\|clostridiales\|ruminococcaceae\|oscillospira\|oscillospira spp..127 | 3.85 | -3.77 | 0.000 | 0.002 |
| bacteria\|bacteroidetes\|bacteroidia\|bacteroidales\|bacteroidaceae\|bacteroides\|bacteroides caccae.6 | 4.67 | 2.27 | 0.006 | 0.027 |
| bacteria\|firmicutes\|negativicutes\|selenomonadales\|veillonellaceae\|veillonella\|veillonella parvula.1 | 5.97 | 2.05 | 0.003 | 0.016 |
| bacteria\|bacteroidetes\|bacteroidia\|bacteroidales\|bacteroidaceae\|bacteroides\|bacteroides ovatus.7 | 38.23 | 1.48 | 0.002 | 0.010 |
| bacteria\|firmicutes\|clostridia\|clostridiales\|lachnospiraceae\|lachnoclostridium\|clostridium lavalense.2 | 4.26 | 2.93 | 0.000 | 0.004 |
| bacteria\|bacteroidetes\|bacteroidia\|bacteroidales\|bacteroidaceae\|bacteroides\|bacteroides eggerthii.1 | 54.76 | 0.91 | 0.006 | 0.026 |
| bacteria\|firmicutes\|clostridia\|clostridiales\|lachnospiraceae\|lachnoclostridium\|clostridium polysaccharolyticum.6 | 1.93 | -2.98 | 0.001 | 0.006 |
| bacteria\|firmicutes\|erysipelotrichia\|erysipelotrichales\|erysipelotrichaceae\|erysipelatoclostridium\|clostridium spiroforme.3 | 14.28 | 2.36 | 0.003 | 0.016 |
| bacteria\|firmicutes\|negativicutes\|selenomonadales\|veillonellaceae\|dialister\|dialister invisus.3 | 41.91 | 2.58 | 0.000 | 0.002 |
| bacteria\|actinobacteria\|actinobacteria\|bifidobacteriales\|bifidobacteriaceae\|bifidobacterium\|bifidobacterium catenulatum.5 | 109.53 | 4.33 | 0.000 | 0.000 |
| bacteria\|firmicutes\|clostridia\|clostridiales\|lachnospiraceae\|lachnoclostridium\|clostridium saccharolyticum.4 | 33.41 | 3.87 | 0.000 | 0.000 |
| bacteria\|firmicutes\|clostridia\|clostridiales\|eubacteriaceae\|eubacterium\|eubacterium rectale.40 | 2.92 | -2.09 | 0.010 | 0.039 |
| bacteria\|firmicutes\|clostridia\|clostridiales\|clostridiaceae\|clostridium\|clostridium spp..53 | 2.44 | -2.84 | 0.013 | 0.048 |
| bacteria\|bacteroidetes\|bacteroidia\|bacteroidales\|porphyromonadaceae\|odoribacter\|odoribacter splanchnicus.3 | 67.30 | -3.70 | 0.000 | 0.000 |
| bacteria\|bacteroidetes\|bacteroidia\|bacteroidales\|bacteroidaceae\|bacteroides\|bacteroides vulgatus.11 | 20.55 | 2.06 | 0.011 | 0.041 |
| bacteria\|firmicutes\|clostridia\|clostridiales\|lachnospiraceae\|lachnoclostridium\|clostridium bolteae.2 | 17.59 | 1.56 | 0.000 | 0.001 |
| bacteria\|bacteroidetes\|bacteroidia\|bacteroidales\|bacteroidaceae\|bacteroides\|bacteroides caccae.8 | 12.22 | 2.98 | 0.001 | 0.004 |
| bacteria\|firmicutes\|clostridia\|clostridiales\|lachnospiraceae\|dorea\|candidatus dorea massiliensis.11 | 9.93 | -2.01 | 0.004 | 0.021 |
| bacteria\|firmicutes\|clostridia\|clostridiales\|clostridiales\|flavonifractor\|clostridium orbiscindens.8 | 105.72 | 3.47 | 0.000 | 0.000 |
| bacteria\|actinobacteria\|actinobacteria\|bifidobacteriales\|bifidobacteriaceae\|bifidobacterium\|bifidobacterium catenulatum.6 | 15.39 | 3.08 | 0.000 | 0.000 |
| bacteria\|firmicutes\|clostridia\|clostridiales\|clostridiales\|flavonifractor\|clostridium orbiscindens.9 | 1.67 | 3.59 | 0.002 | 0.011 |
| bacteria\|bacteroidetes\|bacteroidia\|bacteroidales\|rikenellaceae\|alistipes\|alistipes shahii.2 | 338.56 | -3.58 | 0.000 | 0.000 |
| bacteria\|firmicutes\|clostridia\|clostridiales\|lachnospiraceae\|lachnoclostridium\|clostridium aldenense.2 | 34.51 | 1.58 | 0.011 | 0.041 |
| bacteria\|firmicutes\|clostridia\|clostridiales\|ruminococcaceae\|oscillospira\|oscillospira spp..143 | 13.73 | 2.81 | 0.000 | 0.002 |
| bacteria\|firmicutes\|clostridia\|clostridiales\|eubacteriaceae\|eubacterium\|eubacterium coprostanoligenes.19 | 3.81 | -2.87 | 0.004 | 0.021 |
| bacteria\|firmicutes\|clostridia\|clostridiales\|clostridiaceae\|clostridium\|clostridium spp..57 | 35.70 | 2.47 | 0.000 | 0.002 |
| bacteria\|firmicutes\|clostridia\|clostridiales\|ruminococcaceae\|oscillospira\|oscillospira spp..145 | 17.44 | -2.10 | 0.000 | 0.000 |
| bacteria\|bacteroidetes\|bacteroidia\|bacteroidales\|bacteroidaceae\|bacteroides\|bacteroides dorei.5 | 73.53 | 1.97 | 0.010 | 0.038 |
| bacteria\|firmicutes\|clostridia\|clostridiales\|ruminococcaceae\|oscillospira\|oscillospira spp..147 | 3.93 | -4.08 | 0.000 | 0.000 |
| bacteria\|firmicutes\|clostridia\|clostridiales\|lachnospiraceae\|blautia\|ruminococcus gnavus.20 | 12.52 | 1.89 | 0.010 | 0.038 |
| bacteria\|firmicutes\|clostridia\|clostridiales\|eubacteriaceae\|eubacterium\|eubacterium rectale.44 | 1.41 | -2.73 | 0.012 | 0.044 |
| bacteria\|actinobacteria\|actinobacteria\|bifidobacteriales\|bifidobacteriaceae\|bifidobacterium\|bifidobacterium bifidum.2 | 1.07 | 3.27 | 0.002 | 0.013 |
| bacteria\|firmicutes\|clostridia\|clostridiales\|ruminococcaceae\|subdoligranulum\|subdoligranulum spp..36 | 2.98 | 2.97 | 0.004 | 0.020 |
| bacteria\|firmicutes\|clostridia\|clostridiales\|eubacteriaceae\|eubacterium\|eubacterium coprostanoligenes.20 | 17.82 | -3.92 | 0.000 | 0.000 |
| bacteria\|firmicutes\|negativicutes\|selenomonadales\|veillonellaceae\|megasphaera\|megasphaera elsdenii.2 | 8.25 | 4.80 | 0.000 | 0.003 |
| bacteria\|firmicutes\|clostridia\|clostridiales\|ruminococcaceae\|oscillospira\|oscillospira spp..161 | 2.36 | 3.43 | 0.005 | 0.024 |
| bacteria\|firmicutes\|clostridia\|clostridiales\|ruminococcaceae\|oscillospira\|oscillospira spp..168 | 13.28 | -3.25 | 0.000 | 0.000 |
| bacteria\|firmicutes\|clostridia\|clostridiales\|lachnospiraceae\|eisenbergiella\|eisenbergiella tayi.5 | 3.68 | 4.34 | 0.000 | 0.001 |
| bacteria\|bacteroidetes\|bacteroidia\|bacteroidales\|bacteroidaceae\|bacteroides\|bacteroides thetaiotaomicron.10 | 22.26 | 2.94 | 0.000 | 0.000 |
| bacteria\|firmicutes\|clostridia\|clostridiales\|clostridiaceae\|clostridium\|clostridium spp..65 | 1.39 | 3.05 | 0.001 | 0.007 |
| bacteria\|bacteroidetes\|bacteroidia\|bacteroidales\|rikenellaceae\|alistipes\|alistipes shahii.3 | 5.86 | -3.71 | 0.000 | 0.001 |
| bacteria\|firmicutes\|clostridia\|clostridiales\|ruminococcaceae\|ruminococcus\|ruminococcus sp..48 | 7.68 | 1.76 | 0.009 | 0.037 |
| bacteria\|bacteroidetes\|bacteroidia\|bacteroidales\|bacteroidaceae\|bacteroides\|bacteroides fragilis.5 | 17.38 | 2.53 | 0.005 | 0.021 |
| bacteria\|bacteroidetes\|bacteroidia\|bacteroidales\|bacteroidaceae\|bacteroides\|bacteroides vulgatus.14 | 3.25 | 1.39 | 0.013 | 0.048 |
| bacteria\|firmicutes\|erysipelotrichia\|erysipelotrichales\|erysipelotrichaceae\|holdemanella\|eubacterium biforme.3 | 21.77 | -3.16 | 0.000 | 0.000 |
| bacteria\|firmicutes\|clostridia\|clostridiales\|clostridiaceae\|clostridium\|clostridium sp..163 | 18.54 | -3.14 | 0.000 | 0.002 |
| bacteria\|bacteroidetes\|bacteroidia\|bacteroidales\|bacteroidaceae\|bacteroides\|bacteroides caccae.9 | 22.23 | 2.70 | 0.002 | 0.013 |
| bacteria\|firmicutes\|clostridia\|clostridiales\|lachnospiraceae\|roseburia\|roseburia intestinalis.2 | 7.08 | 2.89 | 0.000 | 0.003 |
| bacteria\|firmicutes\|clostridia\|clostridiales\|lachnospiraceae\|roseburia\|roseburia faecis.33 | 97.63 | 1.64 | 0.010 | 0.040 |
| bacteria\|firmicutes\|clostridia\|clostridiales\|eubacteriaceae\|eubacterium\|eubacterium rectale.48 | 3.80 | -2.76 | 0.003 | 0.014 |
| bacteria\|bacteroidetes\|bacteroidia\|bacteroidales\|bacteroidaceae\|bacteroides\|bacteroides intestinalis.6 | 268.34 | -1.41 | 0.008 | 0.033 |
| bacteria\|firmicutes\|clostridia\|clostridiales\|lachnospiraceae\|blautia\|ruminococcus gnavus.23 | 8.72 | 3.20 | 0.000 | 0.003 |
| bacteria\|bacteroidetes\|bacteroidia\|bacteroidales\|bacteroidaceae\|bacteroides\|bacteroides caccae.10 | 717.81 | 2.23 | 0.001 | 0.004 |
| bacteria\|bacteroidetes\|bacteroidia\|bacteroidales\|bacteroidaceae\|bacteroides\|bacteroides ovatus.9 | 1.89 | 3.63 | 0.009 | 0.037 |
| bacteria\|firmicutes\|clostridia\|clostridiales\|lachnospiraceae\|lachnoclostridium\|clostridium xylanolyticum.7 | 5.90 | -1.82 | 0.002 | 0.012 |
| bacteria\|firmicutes\|clostridia\|clostridiales\|ruminococcaceae\|oscillospira\|oscillospira spp..175 | 11.32 | -3.32 | 0.000 | 0.000 |
| bacteria\|firmicutes\|clostridia\|clostridiales\|clostridiaceae\|clostridium\|clostridium spp..69 | 2.20 | 3.15 | 0.004 | 0.021 |
| bacteria\|proteobacteria\|gammaproteobacteria\|enterobacteriales\|enterobacteriaceae\|escherichia\|escherichia coli.1 | 31.06 | 2.81 | 0.000 | 0.001 |
| bacteria\|firmicutes\|negativicutes\|selenomonadales\|veillonellaceae\|dialister\|dialister invisus.4 | 8.25 | 4.79 | 0.000 | 0.001 |
| bacteria\|firmicutes\|clostridia\|clostridiales\|lachnospiraceae\|blautia\|ruminococcus gnavus.27 | 1.47 | 3.22 | 0.014 | 0.049 |
| bacteria\|bacteroidetes\|bacteroidia\|bacteroidales\|bacteroidaceae\|bacteroides\|bacteroides uniformis.47 | 228.83 | 2.14 | 0.000 | 0.002 |
| bacteria\|firmicutes\|clostridia\|clostridiales\|lachnospiraceae\|blautia\|ruminococcus gnavus.28 | 100.03 | 2.76 | 0.000 | 0.000 |
| bacteria\|firmicutes\|clostridia\|clostridiales\|eubacteriaceae\|eubacterium\|eubacterium rectale.56 | 2.78 | -1.97 | 0.012 | 0.045 |
| bacteria\|firmicutes\|negativicutes\|selenomonadales\|veillonellaceae\|megasphaera\|megasphaera elsdenii.3 | 3.55 | 3.86 | 0.010 | 0.040 |
| bacteria\|bacteroidetes\|bacteroidia\|bacteroidales\|bacteroidaceae\|bacteroides\|bacteroides thetaiotaomicron.13 | 220.56 | 2.77 | 0.000 | 0.000 |
| bacteria\|firmicutes\|clostridia\|clostridiales\|ruminococcaceae\|oscillospira\|oscillospira spp..192 | 3.60 | -3.23 | 0.001 | 0.005 |
| bacteria\|firmicutes\|clostridia\|clostridiales\|ruminococcaceae\|oscillospira\|oscillospira spp..193 | 5.20 | -3.90 | 0.000 | 0.000 |
| bacteria\|firmicutes\|clostridia\|clostridiales\|clostridiales\|intestinimonas\|intestinimonas butyriciproducens.8 | 2.16 | -3.40 | 0.008 | 0.035 |
| bacteria\|bacteroidetes\|bacteroidia\|bacteroidales\|porphyromonadaceae\|butyricimonas\|butyricimonas virosa.16 | 13.54 | -4.06 | 0.000 | 0.001 |
| bacteria\|firmicutes\|clostridia\|clostridiales\|eubacteriaceae\|eubacterium\|eubacterium rectale.58 | 3.00 | 3.19 | 0.000 | 0.001 |
| bacteria\|bacteroidetes\|bacteroidia\|bacteroidales\|bacteroidaceae\|bacteroides\|bacteroides uniformis.54 | 2.36 | 3.73 | 0.002 | 0.012 |
| bacteria\|firmicutes\|clostridia\|clostridiales\|lachnospiraceae\|blautia\|ruminococcus gnavus.31 | 12.59 | 1.80 | 0.009 | 0.037 |
| bacteria\|firmicutes\|clostridia\|clostridiales\|ruminococcaceae\|oscillospira\|oscillospira spp..201 | 4.48 | -2.88 | 0.001 | 0.006 |
| bacteria\|bacteroidetes\|bacteroidia\|bacteroidales\|bacteroidaceae\|bacteroides\|bacteroides faecichinchillae | 71.09 | 3.07 | 0.000 | 0.000 |
| bacteria\|firmicutes\|clostridia\|clostridiales\|ruminococcaceae\|ruminiclostridium\|clostridium methylpentosum.13 | 1.64 | 2.80 | 0.007 | 0.030 |
| bacteria\|bacteroidetes\|bacteroidia\|bacteroidales\|porphyromonadaceae\|butyricimonas\|butyricimonas sp..39 | 79.56 | -4.26 | 0.000 | 0.000 |
| bacteria\|firmicutes\|clostridia\|clostridiales\|ruminococcaceae\|oscillospira\|oscillospira spp..214 | 2.71 | 2.88 | 0.002 | 0.011 |
| bacteria\|bacteroidetes\|bacteroidia\|bacteroidales\|bacteroidaceae\|bacteroides\|bacteroides vulgatus.16 | 49.73 | 1.65 | 0.000 | 0.003 |
| bacteria\|firmicutes\|clostridia\|clostridiales\|ruminococcaceae\|oscillospira\|oscillospira spp..217 | 8.63 | -3.21 | 0.000 | 0.003 |
| bacteria\|firmicutes\|clostridia\|clostridiales\|eubacteriaceae\|eubacterium\|eubacterium coprostanoligenes.24 | 7.91 | -4.27 | 0.000 | 0.001 |
| bacteria\|firmicutes\|clostridia\|clostridiales\|lachnospiraceae\|lachnoclostridium\|clostridium bolteae.5 | 3.27 | 3.16 | 0.000 | 0.003 |
| bacteria\|firmicutes\|clostridia\|clostridiales\|lachnospiraceae\|lachnoclostridium\|clostridium saccharolyticum.10 | 2.39 | -2.78 | 0.002 | 0.012 |
| bacteria\|firmicutes\|clostridia\|clostridiales\|oscillospiraceae\|oscillibacter\|oscillibacter sp..3 | 6.68 | -3.76 | 0.000 | 0.000 |
| bacteria\|actinobacteria\|actinobacteria\|bifidobacteriales\|bifidobacteriaceae\|bifidobacterium\|bifidobacterium bifidum.3 | 4.47 | 3.48 | 0.001 | 0.004 |
| bacteria\|firmicutes\|clostridia\|clostridiales\|lachnospiraceae\|lachnoclostridium\|clostridium saccharolyticum.11 | 1.55 | -3.17 | 0.007 | 0.031 |
| bacteria\|firmicutes\|clostridia\|clostridiales\|clostridiaceae\|clostridium\|clostridium sp..213 | 3.99 | -3.43 | 0.003 | 0.015 |
| bacteria\|firmicutes\|clostridia\|clostridiales\|ruminococcaceae\|oscillospira\|oscillospira spp..228 | 19.34 | -3.48 | 0.000 | 0.000 |
| bacteria\|firmicutes\|clostridia\|clostridiales\|lachnospiraceae\|eisenbergiella\|eisenbergiella tayi.7 | 5.43 | 3.67 | 0.000 | 0.001 |
| bacteria\|firmicutes\|clostridia\|clostridiales\|ruminococcaceae\|oscillospira\|oscillospira spp..233 | 10.58 | 2.57 | 0.000 | 0.002 |
| bacteria\|firmicutes\|clostridia\|clostridiales\|ruminococcaceae\|oscillospira\|oscillospira spp..235 | 7.63 | -3.94 | 0.000 | 0.000 |
| bacteria\|bacteroidetes\|bacteroidia\|bacteroidales\|rikenellaceae\|alistipes\|alistipes finegoldii.13 | 255.78 | 3.00 | 0.001 | 0.006 |
| bacteria\|firmicutes\|clostridia\|clostridiales\|lachnospiraceae\|lachnoclostridium\|clostridium saccharolyticum.12 | 24.20 | -2.04 | 0.002 | 0.011 |
| bacteria\|bacteroidetes\|bacteroidia\|bacteroidales\|bacteroidaceae\|bacteroides\|bacteroides uniformis.61 | 65.69 | 1.64 | 0.009 | 0.038 |
| bacteria\|firmicutes\|clostridia\|clostridiales\|ruminococcaceae\|oscillospira\|oscillospira spp..242 | 2.38 | -2.53 | 0.003 | 0.016 |
| bacteria\|firmicutes\|clostridia\|clostridiales\|clostridiaceae\|clostridium\|clostridium spp..84 | 6.79 | 1.45 | 0.006 | 0.026 |
| bacteria\|bacteroidetes\|bacteroidia\|bacteroidales\|bacteroidaceae\|bacteroides\|bacteroides ovatus.12 | 5.19 | 3.30 | 0.007 | 0.030 |
| bacteria\|bacteroidetes\|bacteroidia\|bacteroidales\|bacteroidaceae\|bacteroides\|bacteroides spp..22 | 1.68 | 2.27 | 0.004 | 0.021 |
| bacteria\|firmicutes\|clostridia\|clostridiales\|lachnospiraceae\|blautia\|ruminococcus gnavus.39 | 6.76 | 2.35 | 0.013 | 0.048 |
| bacteria\|bacteroidetes\|bacteroidia\|bacteroidales\|bacteroidaceae\|bacteroides\|bacteroides spp..23 | 54.64 | 2.07 | 0.000 | 0.000 |
| bacteria\|firmicutes\|clostridia\|clostridiales\|ruminococcaceae\|ruminococcus\|ruminococcus sp..78 | 6.46 | 1.93 | 0.014 | 0.049 |
| bacteria\|bacteroidetes\|bacteroidia\|bacteroidales\|bacteroidaceae\|bacteroides\|bacteroides uniformis.67 | 79.78 | 1.64 | 0.008 | 0.033 |
| bacteria\|bacteroidetes\|bacteroidia\|bacteroidales\|bacteroidaceae\|bacteroides\|bacteroides thetaiotaomicron.18 | 7.45 | 2.40 | 0.000 | 0.002 |
| bacteria\|bacteroidetes\|bacteroidia\|bacteroidales\|bacteroidaceae\|bacteroides\|bacteroides vulgatus.21 | 10.39 | 1.78 | 0.001 | 0.005 |
| bacteria\|bacteroidetes\|bacteroidia\|bacteroidales\|bacteroidaceae\|bacteroides\|bacteroides uniformis.71 | 30.62 | 2.59 | 0.000 | 0.001 |
| bacteria\|bacteroidetes\|bacteroidia\|bacteroidales\|porphyromonadaceae\|paludibacter\|paludibacter spp..61 | 1.55 | -3.34 | 0.012 | 0.045 |
| bacteria\|bacteroidetes\|bacteroidia\|bacteroidales\|bacteroidaceae\|bacteroides\|bacteroides fragilis.11 | 2.89 | 3.18 | 0.010 | 0.040 |
| bacteria\|firmicutes\|clostridia\|clostridiales\|clostridiaceae\|clostridium\|clostridium sp..241 | 4.43 | -3.50 | 0.001 | 0.008 |
| bacteria\|bacteroidetes\|bacteroidia\|bacteroidales\|bacteroidaceae\|bacteroides\|bacteroides dorei.8 | 766.35 | 2.21 | 0.000 | 0.000 |
| bacteria\|firmicutes\|clostridia\|clostridiales\|lachnospiraceae\|roseburia\|roseburia inulinivorans.18 | 8.08 | -2.76 | 0.004 | 0.020 |
| bacteria\|bacteroidetes\|bacteroidia\|bacteroidales\|bacteroidaceae\|bacteroides\|bacteroides caccae.15 | 498.54 | 2.77 | 0.000 | 0.001 |
| bacteria\|firmicutes\|clostridia\|clostridiales\|ruminococcaceae\|oscillospira\|oscillospira spp..259 | 17.45 | -5.85 | 0.000 | 0.000 |
| bacteria\|bacteroidetes\|bacteroidia\|bacteroidales\|bacteroidaceae\|bacteroides\|bacteroides fragilis.13 | 18.98 | 3.51 | 0.000 | 0.001 |
| bacteria\|firmicutes\|clostridia\|clostridiales\|ruminococcaceae\|oscillospira\|oscillospira spp..262 | 1.19 | 2.26 | 0.014 | 0.049 |
| bacteria\|firmicutes\|negativicutes\|selenomonadales\|veillonellaceae\|megasphaera\|megasphaera elsdenii.4 | 5.92 | 4.41 | 0.000 | 0.001 |
| bacteria\|firmicutes\|clostridia\|clostridiales\|ruminococcaceae\|oscillospira\|oscillospira spp..265 | 1.29 | 3.22 | 0.002 | 0.014 |
| bacteria\|bacteroidetes\|bacteroidia\|bacteroidales\|rikenellaceae\|alistipes\|alistipes finegoldii.19 | 25.02 | 2.05 | 0.013 | 0.046 |
| bacteria\|bacteroidetes\|bacteroidia\|bacteroidales\|bacteroidaceae\|bacteroides\|bacteroides uniformis.76 | 1.98 | 2.76 | 0.014 | 0.049 |
| bacteria\|proteobacteria\|betaproteobacteria\|burkholderiales\|sutterellaceae\|sutterella\|sutterella spp..13 | 91.18 | -3.40 | 0.000 | 0.000 |
| bacteria\|firmicutes\|clostridia\|clostridiales\|lachnospiraceae\|lachnoclostridium\|clostridium bolteae.9 | 8.57 | 2.07 | 0.002 | 0.010 |
| bacteria\|firmicutes\|clostridia\|clostridiales\|lachnospiraceae\|lachnoclostridium\|clostridium symbiosum.4 | 2.71 | 3.72 | 0.000 | 0.001 |
| bacteria\|bacteroidetes\|bacteroidia\|bacteroidales\|bacteroidaceae\|bacteroides\|bacteroides uniformis.79 | 3.21 | 2.64 | 0.003 | 0.016 |
| bacteria\|firmicutes\|clostridia\|clostridiales\|lachnospiraceae\|blautia\|blautia sp..8 | 40.41 | 2.16 | 0.000 | 0.003 |
| bacteria\|proteobacteria\|betaproteobacteria\|burkholderiales\|sutterellaceae\|sutterella\|sutterella spp..14 | 8.73 | -4.16 | 0.000 | 0.002 |
| bacteria\|firmicutes\|clostridia\|clostridiales\|eubacteriaceae\|eubacterium\|eubacterium rectale.89 | 3.64 | 2.44 | 0.005 | 0.021 |
| bacteria\|firmicutes\|clostridia\|clostridiales\|ruminococcaceae\|oscillospira\|oscillospira spp..270 | 7.59 | -3.19 | 0.000 | 0.000 |
| bacteria\|bacteroidetes\|bacteroidia\|bacteroidales\|porphyromonadaceae\|paludibacter\|paludibacter spp..67 | 4.23 | -3.44 | 0.001 | 0.006 |
| bacteria\|firmicutes\|clostridia\|clostridiales\|eubacteriaceae\|eubacterium\|eubacterium rectale.96 | 1.41 | 2.13 | 0.008 | 0.034 |
| bacteria\|firmicutes\|clostridia\|clostridiales\|lachnospiraceae\|blautia\|ruminococcus gnavus.44 | 44.61 | 3.19 | 0.000 | 0.002 |
| bacteria\|firmicutes\|clostridia\|clostridiales\|lachnospiraceae\|blautia\|ruminococcus gnavus.45 | 14.77 | 2.52 | 0.000 | 0.000 |
| bacteria\|actinobacteria\|actinobacteria\|bifidobacteriales\|bifidobacteriaceae\|bifidobacterium\|bifidobacterium bifidum.4 | 4.58 | 3.15 | 0.000 | 0.003 |
| bacteria\|firmicutes\|clostridia\|clostridiales\|ruminococcaceae\|ruminococcus\|ruminococcus sp..87 | 6.77 | 3.06 | 0.000 | 0.003 |
| bacteria\|firmicutes\|clostridia\|clostridiales\|ruminococcaceae\|oscillospira\|oscillospira spp..273 | 1.20 | -2.19 | 0.014 | 0.049 |
| bacteria\|firmicutes\|clostridia\|clostridiales\|lachnospiraceae\|blautia\|blautia producta.21 | 1.39 | -2.68 | 0.011 | 0.042 |
| bacteria\|firmicutes\|clostridia\|clostridiales\|ruminococcaceae\|oscillospira\|oscillospira spp..281 | 17.26 | -3.59 | 0.000 | 0.000 |
| bacteria\|firmicutes\|clostridia\|clostridiales\|ruminococcaceae\|oscillospira\|oscillospira spp..289 | 2.45 | -3.08 | 0.000 | 0.003 |
| bacteria\|firmicutes\|clostridia\|clostridiales\|clostridiaceae\|clostridium\|clostridium sp..262 | 35.26 | -3.05 | 0.000 | 0.000 |
| bacteria\|firmicutes\|clostridia\|clostridiales\|lachnospiraceae\|blautia\|blautia producta.22 | 22.09 | -1.98 | 0.000 | 0.003 |
| bacteria\|bacteroidetes\|bacteroidia\|bacteroidales\|bacteroidaceae\|bacteroides\|bacteroides vulgatus.27 | 2.18 | 3.19 | 0.003 | 0.015 |
| bacteria\|firmicutes\|clostridia\|clostridiales\|ruminococcaceae\|faecalibacterium\|faecalibacterium prausnitzii.40 | 18.86 | -1.14 | 0.011 | 0.041 |
